# Microbial eukaryotic predation pressure and biomass at deep-sea hydrothermal vents

**DOI:** 10.1093/ismejo/wrae004

**Published:** 2024-01-13

**Authors:** Sarah K Hu, Rika E Anderson, Maria G Pachiadaki, Virginia P Edgcomb, Margrethe H Serres, Sean P Sylva, Christopher R German, Jeffrey S Seewald, Susan Q Lang, Julie A Huber

**Affiliations:** Department of Oceanography, Texas A&M University, College Station, TX 77843, United States; Department of Marine Chemistry and Geochemistry, Woods Hole Oceanographic Institution, Woods Hole, MA 02543, United States; Biology Department, Carleton College, Northfield, MN 55057, United States; Biology Department, Woods Hole Oceanographic Institution, Woods Hole, MA 02543, United States; Department of Geology & Geophysics, Woods Hole Oceanographic Institution, Woods Hole, MA 02543, United States; Department of Marine Chemistry and Geochemistry, Woods Hole Oceanographic Institution, Woods Hole, MA 02543, United States; Department of Marine Chemistry and Geochemistry, Woods Hole Oceanographic Institution, Woods Hole, MA 02543, United States; Department of Geology & Geophysics, Woods Hole Oceanographic Institution, Woods Hole, MA 02543, United States; Department of Marine Chemistry and Geochemistry, Woods Hole Oceanographic Institution, Woods Hole, MA 02543, United States; Department of Geology & Geophysics, Woods Hole Oceanographic Institution, Woods Hole, MA 02543, United States; Department of Marine Chemistry and Geochemistry, Woods Hole Oceanographic Institution, Woods Hole, MA 02543, United States

**Keywords:** deep-sea hydrothermal vents, microbial eukaryotes, predator–prey interactions, deep-sea food web ecology, protists

## Abstract

Deep-sea hydrothermal vent geochemistry shapes the foundation of the microbial food web by fueling chemolithoautotrophic microbial activity. Microbial eukaryotes (or protists) play a critical role in hydrothermal vent food webs as consumers and hosts of symbiotic bacteria, and as a nutritional source to higher trophic levels. We measured microbial eukaryotic cell abundance and predation pressure in low-temperature diffuse hydrothermal fluids at the Von Damm and Piccard vent fields along the Mid-Cayman Rise in the Western Caribbean Sea. We present findings from experiments performed under *in situ* pressure that show cell abundances and grazing rates higher than those done at 1 atmosphere (shipboard ambient pressure); this trend was attributed to the impact of depressurization on cell integrity. A relationship between the protistan grazing rate, prey cell abundance, and temperature of end-member hydrothermal vent fluid was observed at both vent fields, regardless of experimental approach. Our results show substantial protistan biomass at hydrothermally fueled microbial food webs, and when coupled with improved grazing estimates, suggest an important contribution of grazers to the local carbon export and supply of nutrient resources to the deep ocean.

## Graphical Abstract

**Graphical Abstract:**
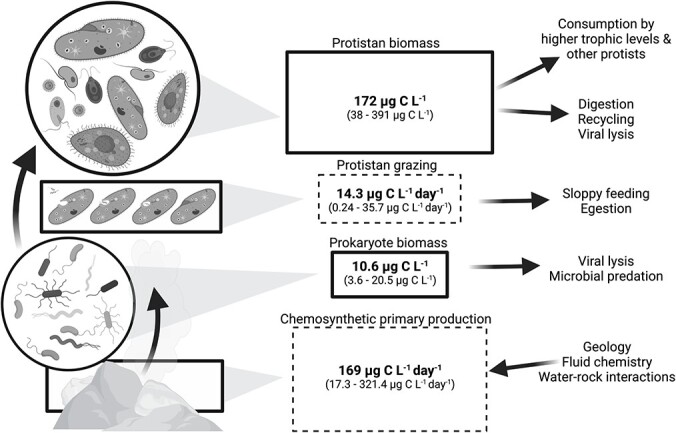

## Introduction

The microbial food web at deep-sea hydrothermal vents is fueled by primary production that is sourced from chemolithoautotrophic microorganisms interacting with diffuse vent fluids. Due to the localized abundance of energy, hydrothermal vent sites support a rich microbial and animal community [1, 2]. Genetic studies have revealed that these sites host highly diverse and distinct bacteria, archaea, viral, and protistan assemblages [3-9]. Unicellular microbial eukaryotes (or protists) are key components of this ecosystem and have an impact on hydrothermal food webs as grazers of local microbial communities [10, 11], parasites [12], or hosts to symbiotic bacteria or archaea [13, 14], as well as a nutritional resource for higher trophic levels (e.g. other protists, mesozooplankton, or invertebrates) [15, 16].

Our understanding of the trophic exchange and flux of nutrients during deep-sea microbial interactions is limited due to the logistical challenges of accurately measuring microbial community interactions *in situ* [17, 18]. The process of collecting vent fluid samples and bringing them shipboard from the deep sea, via Niskin bottle casts or vehicle operations, undoubtedly introduces sampling artifacts due to changes in the pressure and temperature and the chemical environment [19]. Approaches to reduce sampling bias include instrumentation that enables experimentation at the seafloor and the ability to chemically fix organisms at depth, thereby preserving *in situ* metabolic information [8, 17, 20, 21]. Other methods include chambers that can be used to recover deep-sea fluid and the organisms they contain, while retaining *in situ* pressure during shipboard recovery and subsequent experimental processing [22, 23].

Here, we report measurements of protistan grazing activity and biomass from low-temperature diffuse hydrothermal vent fluids collected from two vent fields that are situated 20-km apart at the Mid-Cayman Rise: the Von Damm and Piccard vent fields. Protistan grazing experiments were conducted at both ambient (shipboard) and *in situ* (using isobaric gas-tight chambers [IGTs] [22]) pressure to evaluate how depressurization influences the results of incubations. The resulting findings enabled comparisons between vent fields, vent-to-background environments, and experimental approaches to assess the impact that the local hydrothermal vent geochemistry has on microbial biomass and grazing pressure. This study complements previous molecular-based observations of the highly diverse and spatially distinct protistan populations found at the Mid-Cayman Rise [6] with, to our knowledge are, the first assessments of deep-sea hydrothermal vent protistan cell concentration and biomass. The results reported here contribute to ongoing efforts to quantify deep-sea hydrothermal food web interactions, especially those involving microbial eukaryotes.

## Materials and methods

### Fluid collection at the Mid-Cayman Rise

Samples and experiments were collected and executed during cruise AT42-22 (doi: 10.7284/908847) aboard the research vessel (RV) *Atlantis* with the remotely operated vehicle (ROV) *Jason* in January–February 2020 at the Von Damm (2300 m; 18° 23′ N, 81° 48′ W) and Piccard (5000 m; 18° 33′ N, 81° 43′ W) hydrothermal fields located along the Mid-Cayman Rise (Fig. S1). Fluids for shipboard grazing experiments and biogeochemistry were obtained in 10-L volume bags (Kynar, Keika Ventures; polyvinylidene fluoride) using the Hydrothermal Organic Geochemistry (HOG) sampler mounted on ROV *Jason* [24]. Between 4 and 10 L of vent fluid was collected and filtered through a 47-mm polyethersulfone filter (Millipore) with a pore size of 0.2 μm to capture all microorganisms. The filter was preserved with RNAlater (Ambion) at the seafloor for molecular analysis of microbial communities [8]. Fluids for experiments conducted at *in situ* pressure and parallel geochemical measurements were collected with IGTs [22], which filled at a rate of ~1 ml sec^−1^ (Fig. S1D). Shipboard, dissolved hydrogen gas and methane concentrations were determined by gas chromatography, and pH_25°C_ was measured at room temperature with a combination Ag/AgCl reference electrode. Magnesium was measured in a shore-based laboratory by ion chromatography on stored 30-ml fluid samples. Geochemical measurements from this study were also previously reported [6].

Non-vent samples were collected from within the overlying nonbuoyant hydrothermal plume at each site and from background seawater via CTD-mounted Niskin bottles. Plume samples were identified using *in situ* CTD sensors to detect the presence of hydrothermal influence in real time (backscatter and temperature) above each vent field. Background seawater samples were collected outside of the influence of the hydrothermal vent at approximately the same depth as the vent sites (~2350 and ~4950 m; Table S1).

### Microeukaryote grazing experiments

Protistan grazing experiments (or fluorescently labeled prey [FLP] uptake experiments) were conducted as described previously [25, 26], by using fluids from the Von Damm and Piccard vent fields, their respective buoyant plumes, and background seawater collected at depths appropriate for each site (*n* = 14; Table 1). Most experiments were performed shipboard (9 experiments at ambient pressure), and a subset were carried out at *in situ* pressures in IGTs for comparison (5 experiments at *in situ* pressure, 1 experiment was not countable; see Table 1). For all grazing experiments, FLP, consisting of 5-(4,6-dichlorotriazinyl) aminofluorescein–stained and heat-killed *Hydrogenovibrio* [27, 28], was introduced as the analog prey. For complete details on creating FLP see Supplemental Information and previously published work (similar to [10]).

**Table 1:** Experiments conducted at the Mid-Cayman Rise.^a^

**Vent field**	**Site name**	**Fluid origin**	**Fluid ID**	**Experiment condition**	**Abundance (cells ml** ^**−1**^ **)**				**FLP grazer** ^**−1**^ **hr** ^**−1**^	**Grazing rate (cells consumed ml** ^**−1**^ **hr**^**−1**^**)**	**Clearance rate (mL grazer** ^**−1**^ **hr** ^**−1**^ **)**	**Specific grazing rate (Prokaryotes grazer** ^**−1**^ **hr**^**−1**^**)**	**Bacteria turnover (% removed prokaryotes day** ^**−1**^ **)**	**Temperature (°C)**
					**Eukaryote**		**Prokaryote**							
					**Average**	**Min/max**	**Average**	**Min/max**						
**Von Damm**	Background	CTD002	Niskins 8–10	Ambient	91.838	69.97/113.7	3.79 × 10^4^	1.85/5.65 × 10^4^	0.178	127.07	3.70 × 10^−5^	1.38	8.0	4.2
	Plume	CTD001	Niskin 2	Ambient	157.77	55.98/284.4	1.65 × 10^4^	1.39/1.91 × 10^4^	0.316	24.03	9.24 × 10^−6^	0.15	3.5	4.2
	Mustard Stand	J2–1243	LV17	Ambient	259.77	230.9/288.6	5.67 × 10^4^	4.24/7.10 × 10^4^	bd	bd	bd	bd	bd	108.0
	Old Man Tree	J2–1238	IGT4	*In situ*	349.86	—	—	—	0.226	1050.38	4.22 × 10^−5^	3.00	35.0	121.6
	Ravelin #2	J2–1238	LV13a	Ambient	409.33	335.9/482.8	—	—	0.208	116.86	4.01 × 10^−6^	0.29	3.9	94.0
	Ravelin #2	J2–1244	IGT4	*In situ*	944.62	—	—	—	1.264	15867.10	2.40 × 10^−4^	16.80	540.0	98.2
	Ravelin #2	J2–1244	IGT5	*In situ*	629.74	—	—	—	bd	bd	bd	bd	bd	98.2
	Shrimp Hole	J2–1244	LV13	Ambient	385.72	377.8/393.6	4.20 × 10^4^	3.88/4.51 × 10^4^	bd	bd	bd	bd	bd	21.0
	X-18	J2–1235	LV23 & Bio5	Ambient	314.87	209.9/419.8	1.11 × 10^5^	10.9/1.14 × 10^5^	0.105	1166.28	3.32 × 10^−5^	3.70	25.0	48.0
**Piccard**	Plume	CTD004	Niskin 10	Ambient	79.301	55.98/112	5.14 × 10^4^	4.68/5.61 × 10^4^	0.322	44.26	1.10 × 10^−5^	0.56	2.1	4.5
	Lots 'O Shrimp	J2–1241	LV24	Ambient	230.91	230.9/230.9	5.39 × 10^4^	2.62/9.03 × 10^4^	bd	bd	bd	bd	bd	36.0
	Shrimpocalypse	J2–1240	LV13	Ambient	454.82	—	2.39 × 10^5^	10.90/3.22 × 10^5^	0.941	6006.91	5.54 × 10^−5^	13.21	60.0	85.0
	Shrimpocalypse	J2–1240	IGT3	*In situ*	384.84	—	2.39 × 10^5^	10.90/3.22 × 10^5^	1.008	17284.68	1.90 × 10^−4^	44.91	170.0	85.0
	Shrimpocalypse	J2–1240	IGT7	*In situ*	524.79	—	2.39 × 10^5^	10.90/3.22 × 10^5^	n/a	n/a	n/a	n/a	n/a	85.0

a Includes 9 from Von Damm and 5 from Piccard of 14 total grazing assays; 5 were conducted in isobaric gas tight (IGT) chambers at in situ pressure. Of the IGT experiments, 4 of 5 were usable for grazing experiments; 1 experiment was only used for eukaryote cell abundance. Temperature reflects the highest recorded temperature at time of fluid collection. Prokaryotic cell concentrations were derived from discrete fixed samples from the same fluid, while the reported eukaryotic cell concentrations are derived from the T0 grazing experiment time points. When prokaryote cell abundances were not countable (due to mineral precipitation in the sample), an average prokaryotic cell ml^−1^ was used for downstream calculations (7.11 × 10^4^ cell ml^−1^). Absent grazing rates include those with a negative slope, and percentage prokaryote turnover shows the relative top-down (higher percentage) to bottom-up pressures on the microbial communities, based on grazing rate and cell concentrations. bd, indicates not detected or below detection limit for grazing rate; n/a indicates grazing experiment was not countable; — indicates no value is available.

^*^For prokaryote cells per ml that were not countable, an average across all vents was taken and used for calculations

#### Incubations conducted at ambient pressure

Large-volume bags filled with vent fluid using the HOG sampler were subsampled into 2–3 acid-rinsed and clean bags (polyvinylidene fluoride) at volumes ranging from 1.5 to 2 L (volume and experimental replicates varied based on available water budget). Each shipboard grazing experiment was conducted in duplicate or triplicate (where all treatment volumes were the same; Table S2) and kept at ~22°C for the incubations. To remove microbial predators from control treatments, fluid was filtered through a 0.8-μm porosity filter in duplicate (0.5–1 L). Immediately after the experimental and control fluids were distributed, thoroughly mixed FLP was introduced into each treatment to an FLP concentration that was 20%–25% of the *in situ* prokaryotic community. The incubations were gently mixed and an initial time point, T0, was taken. For each time point, 200- or 20-ml fluid was preserved from the experimental and control treatments, respectively, with chilled formaldehyde (1% final concentration) and stored in darkened amber bottles (20 ml for controls) at 4°C until processing. Less volume (20 ml) was taken from the control treatments, in which only FLP were counted, while a larger volume (200 ml) was taken from the experimental treatments, as required to capture the protistan biomass. Target time points ranged from 0 to 40 minutes (T0, T10, T15, T20, and Tf at 40 minutes); in some cases, T20 time points were not taken due to constraints on recovered hydrothermal fluid volume (Table S2). Following the final time point (Tf), all of the remaining fluid from the shipboard experiments was filtered into a Sterivex filter (porosity of 0.2 μm [Millipore]), preserved with RNAlater, and frozen at −80°C for molecular analysis. These samples represent the community of protists at the end of the shipboard incubations (T40 or Tf).

#### Incubations conducted at *in situ* pressure

Before each IGT sampler was deployed, the dead volume was filled with 0.2-μm filtered background deep seawater, and a Teflon O-ring was added to the sample chamber to enhance mixing of collected fluid and injected amendments. ROV *Jason* positioned the IGT inlet with a co-located temperature probe to collect diffuse vent fluid. Shortly after ROV recovery, a titanium piston separator was affixed to the IGT [similar to 23] to facilitate the introduction of FLP and collect subsamples for grazing experiment time points without rupturing cells by eliminating the need for fluids to pass through the small opening of a pressure retaining sample valve.

Each IGT-based experiment was maintained at *in situ* pressure (Table 1) for the duration of the incubation using a high-performance liquid chromatography pump to compensate for pressure loss during subsampling (also see [23]). FLPs were premixed at a final volume of 8 ml to add to the 150-ml volume of the IGT samples, and the final FLP concentration was 1 × 10^4^ cells ml^−1^. After agitation of the IGT chamber to gently mix the collected vent fluid samples with the added FLPs, an initial (T0) time point was taken by moving the sample into the pressure separator and then emptying a 30-ml sample into amber bottles with chilled formaldehyde (final concentration 1%). Time points were planned for 0, 10, 20, and 40 minutes, but time constraints meant that time points were often taken at irregular intervals (compared to the shipboard incubation sample intervals; Table S2). Unlike the experiments conducted at ambient pressure, for the IGT experiments the samples for molecular analysis were not available at the end of the experiments, due to limited sample volume.

Control treatments concurrent with the IGT samples from vents were not feasible. IGT control treatments were conducted separately by filling IGTs with deep-sea background seawater that had been collected via Niskin bottles on a CTD rosette. Before being placed in the IGT chamber, this background seawater was filtered through a 0.8-μm filter to remove protistan grazers. Then the IGT chamber was pressurized to *in situ* conditions (3000–6000 psi), FLP were added, and the FLP experimental procedure was replicated.

Since opportunities for biological replicate incubations were limited (only 2 [Table S2]), technical replicate cell counts were completed to provide additional confidence in our findings (repeat microscopy counts). Results from technical replicates are reported in the Supplementary Information.

#### Considerations for comparing experiments performed at ambient and deep-sea pressure

To ensure that the sample fluid collected for the ambient and *in situ* pressure experiments originated from the same location, ROV *Jason* placed the sample fluid intakes for the HOG and IGT samplers as close together as possible. Using both real-time video feeds of the diffuse fluid flow and temperature indications, fluids were collected with both devices in succession (Fig. S1). We also prioritized sampling the same location for both the ambient and *in situ* experiments; however, in several experiments the samples were not usable due to leakage of fluid or loss of *in situ* pressure (Ravelin #2 and Shrimpocalypse; Table 1).

Comparisons between IGT- and shipboard-conducted experiments were limited due to the differences in capabilities of each sampling approach, fluid volume capacity, and ability to perform replicate experiments. For IGT experiments, the total sample volume was 150 ml, and running experimental replicates concurrently was not possible. On the other hand, shipboard experiments, at ambient pressure, ranged in total volume from 1.5 to 2 L, and duplicates or triplicates were run concurrently (Table S2). To partially account for these differences, cell enumeration was repeated from the IGT experiments to serve as technical replicates (Supplementary Information). Our interpretations are supported by consistent trends observed at both the Von Damm and Piccard vent fields (same trends at separate vent fields) and similar findings from a previous study [10]. Further, we determined that statistical comparisons were largely inappropriate due to the overall differences in each experimental set up.

### Processing grazing experiment samples

Formaldehyde-fixed samples (final concentration 1%) were kept in the dark and at 4°C until analysis for both prokaryotic and eukaryotic counts. To determine *in situ* microbial cell concentrations, between 1 and 10 ml of the sample fluid was filtered onto 0.2-μm black polycarbonate filters to concentrate prokaryotic cells (bacteria and archaea, or the microbial prey population) and counted under epifluorescence (blue/cyan filter for 4',6-diamidino-2-phenylindole [DAPI]–stained cells). Similarly, 2–5 ml of the grazing experiment control samples were filtered onto 0.2-μm black polycarbonate filters and counted under the fluorescein isothiocyanate filter to ensure the number of FLP did not change for the duration of the experiment. Samples for all grazing treatments were filtered onto 0.8-μm black filters to concentrate the microeukaryote population (volumes ranged between 100 and 200 ml) and stained with a DAPI solution (final DAPI concentration ∼10 μg ml^-1^). Filters for DAPI and fluorescein isothiocyanate were used to count the number of nano- (<20 μm) and micro- (≥20 μm) eukaryotic cells observed and the number of FLP inside each eukaryotic cell (by switching filter sets back and forth). This approach enabled the enumeration of the total number of eukaryotic cells per milliliter and the number of ingested FLP in each cell. A minimum of 30 fields of view were counted for each sample at 100× magnification. Eukaryotic cells were distinguished from other DAPI-stained debris by noting the presence of a nucleus or eukaryote-like cell morphologies (e.g. flagella, cilia, or organelles).

#### Quantifying protistan predation and biomass

Microscopy counts revealed the number of FLP ingested per eukaryotic cell, concentration of bacteria and archaea, and concentration of microbial eukaryotes. For each grazing assay, the average number of FLP ingested by eukaryotes versus incubation time was determined. Across replicates (experiments at ambient pressure only), the mean number of FLP ingested per total eukaryotes observed and the standard mean error was calculated. Due to the small volume of the IGT experiments and the observation that the final IGT time point (Tf) varied drastically from other time points, the IGT Tf samples were removed before estimating the slope. The slope of the best fit line equates to the number of FLP consumed by a protistan grazer every minute (Table 2). The clearance rate (ml grazer^−1^ hr^−1^) and grazing rate (grazing rate: cells consumed ml^−1^ hr^−1^) were also calculated by including the estimated FLP concentration at T0 and cell abundances for prokaryotes and eukaryotes (Table 2). Grazing experiments that resulted in negative slopes were interpreted as “undetected” or “below the detection limit” grazing; these experiments are presented as 0 in the results. Table 2 summarizes the equations used and related references for quantifying grazing impact.

**Table 2:** Equations used for grazing rate estimates and determination of cell carbon content.^b^

**Term**	**Units**	**Equation**	**Description**	**References**
Slope	FLP grazer^−1^ min^−1^	$ y = mx + b $	Slope of best fit line determined by plotting grazing experiment time by number of FLP counted per protistan grazer counted.	Sherr and Sherr, 1993; Caron 2001; Unrein et al. 2007
Clearance rate	mL grazer^−1^ hr^−1^	$\mathrm{Clearance}\ \mathrm{rate}\ \mathrm{mL}\ {\mathrm{grazer}}^{-1}\ {\mathrm{hr}}^{-1}=\frac{m\times 60\ \mathrm{minutes}}{\mathrm{FLP}\ {\mathrm{ml}}^{-1}}$	Volume that a protistan grazer can consume within an hour. FLP ml^−1^ variable is concentration of FLP at T0.	Sherr and Sherr, 1993; Caron 2001; Unrein et al. 2007
Specific grazing rate per hour	Prokaryotes grazer^−1^ hr^−1^	$\mathrm{Specific}\ \mathrm{grazing}\ \mathrm{rate}\ {\mathrm{grazer}}^{-1}\ {\mathrm{hr}}^{-1}=\left(\mathrm{Clearance}\ \mathrm{rate}\ \mathrm{mL}\ {\mathrm{grazer}}^{-1}\ {\mathrm{hr}}^{-1}\right)\times \left(\mathrm{Prokaryotes}\ {\mathrm{ml}}^{-1}\right)$	Numer of prokaryotic cells that a protistan grazer can consume in an hour.	Sherr and Sherr, 1993; Caron 2001; Unrein et al. 2007
Grazing rate	Cells consumed ml^−1^ hr^−1^	$\mathrm{Cells}\ \mathrm{consumed}\ {\mathrm{ml}}^{-1}{\mathrm{hr}}^{-1}=\left(\mathrm{Specific}\ \mathrm{grazing}\ \mathrm{rate}\ {\mathrm{grazer}}^{-1}\ {\mathrm{hr}}^{-1}\right)\times \left(\mathrm{Eukaryote}\ {\mathrm{cells}}^{-1}\right)$	Number of prokaryotic cells ${\mathrm{ml}}^{-1}$ that protistan grazer population can consume in an hour. Also referred to as grazing rate.	Sherr and Sherr, 1993; Caron 2001; Unrein et al. 2007
Grazing rate	Cells consumed ml^−1^ day^−1^	$\mathrm{Cells}\ \mathrm{consumed}\ {\mathrm{ml}}^{-1}{\mathrm{day}}^{-1}=\left(\mathrm{Specific}\ \mathrm{grazing}\ \mathrm{rate}\ {\mathrm{grazer}}^{-1}\ {\mathrm{hr}}^{-1}\right)\times \left(\mathrm{Eukaryote}\ {\mathrm{cells}}^{-1}\right)\times \frac{24\ \mathrm{hrs}}{1\ \mathrm{day}}$	Number of prokaryotic cells in a ml that protistan grazer population can consume in a day. Also referred to as grazing rate.	Sherr and Sherr, 1993; Caron 2001; Unrein et al. 2007
Bacteria turnover	% removed prokaryotes day^−1^	$\mathrm{Bacteria}\ \mathrm{turnover}\%{\mathrm{day}}^{-1}=100\times \frac{\mathrm{Cells}\ \mathrm{consumed}\ {\mathrm{ml}}^{-1}{\mathrm{day}}^{-1}}{\mathrm{Prokaryote}\ {\mathrm{ml}}^{-1}}$	Percent of prokaryotic population that protistan grazers consume in a day.	Sherr and Sherr, 1993; Caron 2001; Unrein et al. 2007
Biovolume	μm^3^	$\mathrm{Biovolume}=\left(\frac{\pi }{6}\right)\times{d}^2\times h$	Estimation of cell biovolume (or volume), where cell is a prolate spheriod shape. The variable *h* is largest cell dimension, and *d* is cross section of *h*.	Hillebrand et al. 1999; Pernice et al. 2015
Carbon conversion by biovolume	pg C cell^−1^ (mixed protist community without diatoms)	$\mathrm{pg}\ \mathrm{C}\ {\mathrm{cell}}^{-1}=0.216\times{\mathrm{biovolume}}^{0.939}$	Using known carbon to volume relationship across an assortment of microbial eukaryotic species, except for diatoms, this equation estimates carbon content of a cell based on volume.	Menden-Duer & Lessard 2000; Pernice et al. 2015

^a^Columns include the term most closely associated with the equation, units of measure, the equation, a description of usage and input variables, and relevant citations. Table S3 is an expanded version of this table, with additional columns associated with R code.

Eukaryotic cell biomass was determined using cell abundance from each T0 time point and estimating carbon content of individual cells. During microscopy counts, Zeiss image processing software was used to determine the “height” and “width” of preserved cells, where height equates to the longest dimension and width equals the longest cross section [29]. Based on these dimensions, we estimated cell biovolume from a random assortment of experimental time points; the biovolume of each cell was determined (μm^3^) based on equations from Pernice et al. [30] and Hillebrand et al. [31], which are also listed in Table 2. Biovolumes were converted to carbon cells^−1^ using carbon conversion rates from Meden-Deuer and Lessard [32] (Tables 2, S3, and S4). This is a field standard practice whereby the carbon conversion factor for protistan biovolume was determined by using a mixed assemblage of protistan species in culture (excluding diatoms). We considered estimates from Meden-Deuer and Lessard [32] to represent the overall range of likely carbon content for heterotrophic cells, assuming that protistan cells captured in our samples are largely heterotrophic. We acknowledge that these estimates are based on heterotrophic species in culture, which are likely physiologically distinct from cells originating from deep-sea vents. In order to estimate the amount of carbon biomass potentially consumed by microbial eukaryotic grazers, we used the carbon conversion rate of 86 fg C cell^−1^ and the previously published estimates for chemosynthetic primary production (17.3–321.4 μg C L^−1^ day^−1^) [33].

### Amplicon sequence survey

Filters retrieved from ROV *Jason* (representing the *in situ* community) and from the final time point of only the shipboard grazing assays were processed identically. RNA was extracted from frozen filters (stored in RNAlater) as amplicon sequences originating from extracted RNA are more likely to represent metabolically active cells, rather than inactive cellular material that may have sunk from above. The filter was first separated from the RNAlater and distributed into tubes with a lysis buffer (Qiagen 1 053 393). The RNAlater was centrifuged for 15 minutes at 16000 × g, and the supernatant was removed. Lysis buffer was added on top of any cellular materials collected, vortexed, and then combined with the filter. The filter and lysis buffer solution was vortexed thoroughly with RNAase-free silica beads. The lysis buffer was then separated from the beads and filter material with a syringe and processed using the Qiagen RNeasy extraction kit (Qiagen 74 104), which included an inline RNAse-free DNase removal step (Qiagen 79 256). Total RNA was reverse transcribed to cDNA and amplified with V4-specific primers [34]. MiSeq 2 × 300–bp paired-end sequencing was performed at the Keck Sequencing Facility at the Josephine Bay Paul Center Marine Biological Laboratory.

Amplicon sequences were processed using QIIME2 (version 2021.4) [35] as described previously [34]. First, sequences were filtered for quality control and primers were removed using cutadapt (error rate, 0.1; minimum overlap, 3 bps [36]). Amplicon sequence variants (ASVs) were then determined using DADA2 [37] in QIIME2. First, paired-end reads were truncated at 260 and 225 bp for the forward and reverse reads, respectively. Then errors in the sequences were estimated (max-ee [maximum number of expected errors] = 2) and chimeric sequences were removed (pooled method). Reference ASVs were assigned taxonomies using the PR2 database (v 4.14; [38, 39]). ASVs serve to approximately represent the species- or strain-level designation. For this analysis, we focused on the microeukaryotic population, removing sequences assigned to prokaryotes or *Metazoa*. Similar to a previously reported approach [6], ASVs were categorized by their distribution, as either vent only or cosmopolitan: vent-only ASVs were found only in vent samples, while cosmopolitan ASVs were found throughout vent, plume, and background samples.

*In situ* and Tf samples were compared to subsets for taxa that may have been enriched within each grazing experiment. An ASV that was present in both *in situ* and associated Tf samples was considered a member of the captured protistan community. To compare the community that was present *in situ* with the community from the grazing incubations, input ASV counts were center–log ratio transformed ahead of principle component analysis. If the total number of sequences and/or ASVs within the group increased, then the taxonomic group was considered to be enriched. In another approach to determine which taxa may be enriched across the vent sample types, we employed the corncob analysis [40], which models the relative and differential abundances of the ASVs as a linear function of vent versus non-vent habitats. Used with the parametric Wald test, corncob allowed us to test the hypothesis that a given ASV will change significantly across the parameters. Positive coefficients indicated that the taxonomic group was enriched at the family level in vent samples compared to non-vent samples.

### Data availability

Intermediate data products and required code to reproduce results can be found at https://shu251.github.io/midcayman-rise-microeuk/. Raw sequence data are available through the NCBI SRA BioProject accession number PRJNA802868.

## Results

### Fluid geochemistry

The Von Damm and Piccard hydrothermal vent fields at the Mid-Cayman Rise are located at different depths, 2350 and 4950 m, respectively, where Piccard is the deepest known hydrothermal vent field [41, 42]. At the time of sample collection, low-temperature diffuse fluid from the Von Damm vent field ranged between 12°C and 129°C, while temperatures at Piccard were between 19°C and 85°C (Table 1; Table S1). Vent fluids from Von Damm have higher concentrations of methane than fluids from Piccard. Piccard vent fluids are more acidic and have highly variable amounts of dissolved hydrogen (Table S1).

### Microbial biomass

**Figure 1:**
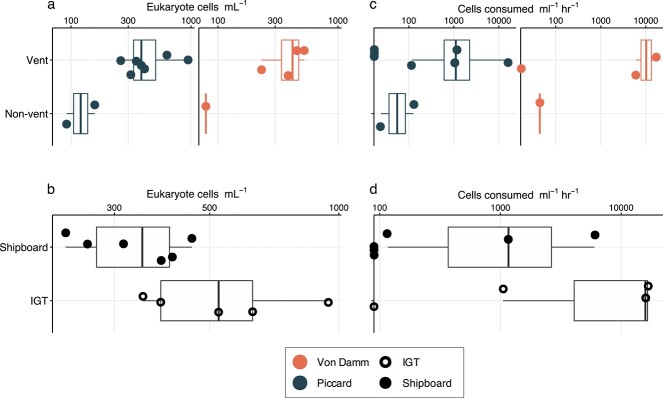
Eukaryotic cell abundance (a and b) and grazing (c and d) results from the Mid-Cayman Rise. Each boxplot outlines the first and third quartiles (lower and upper hinges of the box), and the thicker line in the middle corresponds to the median. Whiskers extending beyond each box show the range of the smallest and largest values. Boxplots are overlaid with the actual values for cell abundances and grazing rates, which are also listed in Table 1. (a) Comparison of eukaryote cells ml^−1^ (log scale) at time zero (Tf) by vent field (Von Damm at left; Piccard at right) and vent habitat type, where vent includes results from all sites of active diffuse flow and non-vent includes plume and deep background seawater. (b) Cell abundances from each vent site are also shown by experimental approach, where shipboard denotes results from grazing experiments conducted at ambient pressure and IGT corresponds to experiments run at in situ pressure. (c) Protistan grazing rates across each vent field and vent versus non-vent environments. Results are expressed as the number of cells consumed by protistan predators ml^−1^ hr^−1^ (log scale). (d) Grazing experiment results from vent sites only are shown again, but grouped by experimental approach (shipboard versus IGT). Symbol color denotes vent field (black symbols in B and D include both Von Damm and Piccard), filled-in circles are derived from shipboard experiments or samples (ambient pressure), and circle outlines represent results from IGT experiments (in situ pressure).

Cell abundances for both bacteria and archaea (prokaryotes) and eukaryote populations shared a similar trend where the highest concentration (cell ml^−1^) was found within diffuse vent fluids, followed by the plume and background environments (Fig. 1a, Table 1). Non-vent (plume and background) prokaryote cell concentrations averaged 3.5 × 10^4^ cells ml^−1^, while concentrations within diffuse vent fluids averaged 1.4 × 10^5^ cells ml^−1^. Prokaryotic cell concentrations within Piccard diffuse fluid (average of 1.9 × 10^5^ cell ml^−1^) were higher than those at Von Damm (average of 7.0 × 10^4^ cells ml^−1^; Table 1).

Eukaryote cell concentrations within the background and plume environments averaged 1.1 × 10^2^ cells ml^−1^. Within diffuse vent fluids, eukaryotic cell abundances were higher than those in non-vent environments, averaging 3.7 × 10^2^ cells ml^−1^. The eukaryotic cell concentrations in the Piccard and Von Damm vent fields were similar, averaging 4.0 × 10^2^ and 3.2 × 10^2^ cells ml^−1^, respectively (Fig. 1a, Table 1). The average eukaryotic cell concentrations derived from samples collected with IGTs, and thus maintained at *in situ* pressure, were slightly higher than those for samples collected with the HOG sampler and then used for shipboard experiments: 4.5 × 10^2^ versus 3.3 × 10^2^ cells ml^−1^ (Fig. 1b). Values reported here include the total number of protists counted (both nano- and microsize classes captured on 0.8-μm filters); results from eukaryotic cell counts separating nano-, micro-, and total (nano + micro) size classes are reported in Fig. S2. Because the process of fixation can shrink cell volume [43, 44], and depressurization may have had an impact on cell integrity, our distinctions of micro- versus nanoplankton size classes may not be accurate. Additional supporting evidence for these distinctions can be found in the Supplemental Information. The majority of our downstream results consider the total microeukaryote population.

The average biovolumes were 773 μm^3^ for protists counted outside vent fluid and 3208.9 μm^3^ for protistan cells found within vent fluid (Table S4; [31]). Biovolume derived from shipboard results averaged only 1976 μm^3^, compared to more than 4400 μm^3^ from the IGT results (the average across IGT and shipboard results was used to estimate C cell^−1^). Using a field standard carbon conversion rate of 0.216 pg C cell^−1^ volume^0.939^ [32], we determined a putative pg C cell^−1^ value for the vent- and non-vent–associated cell abundances. The non-vent background seawater microeukaryote cell carbon factor was determined to be 109.2 pg C cell^−1^, while the cellular carbon content within diffuse vent fluids averaged to 400.8 pg C cell^−1^ (Table S4). These results equate to an estimated total carbon pool of 12.9 μg C L^−1^ outside diffuse vent fluids and 172 μg C L^−1^ within hydrothermal vent fluids (Table 3). The range of carbon biomass estimates by experiment are also reported in Fig. S3.

**Table 3:** Estimated carbon biomass of the protistan population based on different locations (category), such as hydrothermal vent versus non-vent environment, each vent field, or ambient versus in situ pressure conditions.^a^

	**Average carbon biomass (μg C L** ^**−1**^**)**	**Maximum carbon biomass (μg C L** ^**−1**^**)**	**Minimum carbon biomass (μg C L** ^**−1**^**)**
Habitat type comparison			
Non-vent	12.9	17.2	8.7
Vent	172.2	391.8	38.1
Vent field comparison			
Piccard (vent only)	165.4	217.7	95.8
Von Damm (vent only)	175.6	391.8	38.1
Comparison of experimental approach			
Shipboard (vent only)	127.2	188.7	38.1
IGT (vent only)	224.9	391.8	145.1

a Values reported are the average, minimum, and maximum μg of carbon L^−1^, which were determined by multiplying the pg C cell^−1^ (Table 2) by the eukaryotic cell abundances (Table 1; Fig. 1a). Data are grouped by the central comparisons relevant to the main text.

### Protistan grazing

**Figure 2:**
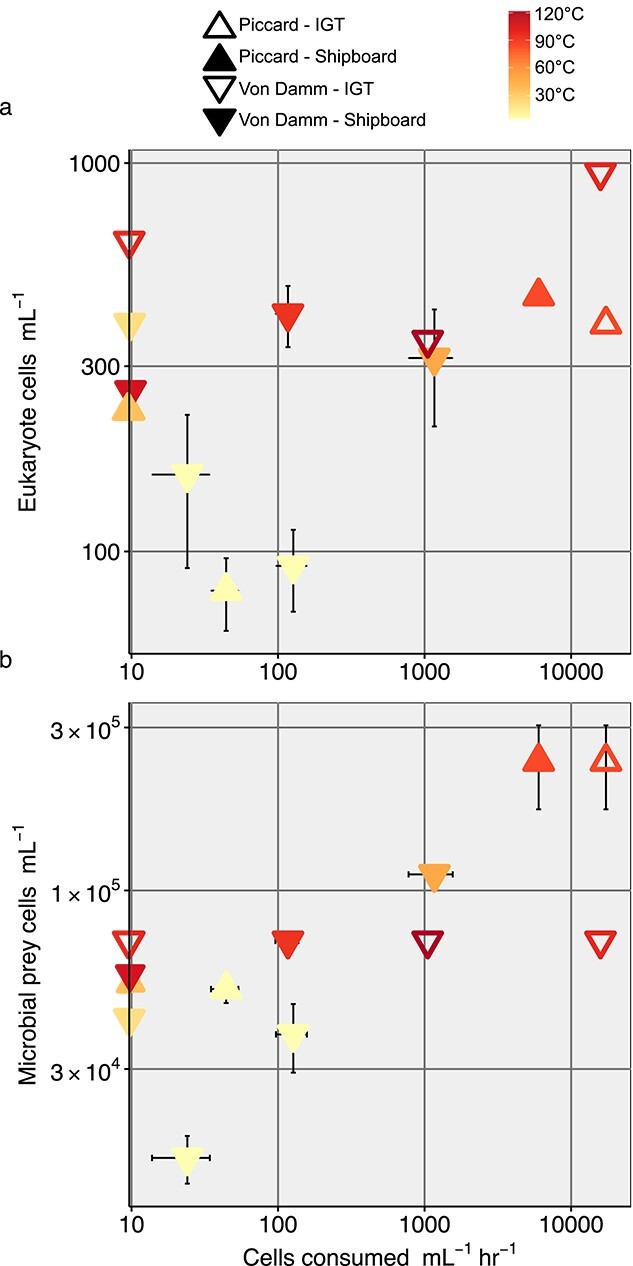
Grazing rates (cells consumed ml^−1^ hr^−1^), along the x-axis, are shown with (a) eukaryote cells ml^−1^ and (b) prokaryote cells ml^−1^ along the y-axes. Symbol color denotes the temperature of fluid at time of sample collection (°C). Filled in triangle symbols are derived from shipboard experiments conducted at ambient pressure, while triangle outlines represent results from IGT experiments performed under in situ pressure. Error bars represent the standard mean error for the cell counts (y-axes) or grazing rate (x-axis). All values are also reported in Table 1.

Based on the observed number of FLP consumed by protistan grazers throughout each experiment, we determined a best fit line for which the slope represents the average number of FLP consumed by protistan grazers per minute ([45]; Tables 2, S2, and S3; Fig. S4). When the slope of the line was negative, grazing was considered undetected or below detection and replaced with a zero value (Table 1). Since eukaryotic cell abundance decreased over time in these experiments (Fig. S5), zero values were not included in reported averages but are included in Figs. 1 and 2. Eukaryotic cells ml^−1^ dramatically decreased in the final time point for each IGT experiment (Tf), warranting the removal of this time point, due to bottle effects (Fig. S5). Results from control experiments were considered stable over time (Supplementary Information; Fig. S6).

The average grazing rate for experiments conducted with diffuse vent fluid was 6.9 × 10^3^ cells consumed ml^−1^ hr^−1^ (minimum, 116.9; maxium, 1.7 × 10^4^ cells consumed ml^−1^ hr^−1^), which was higher than the rate in non-vent samples, where grazing rate averaged 65 cells consumed ml^−1^ hr^−1^ (minimum, 24; maxium, 127 cells consumed ml^−1^ hr^−1^; Fig. 1c). Between the two vent fields, grazing rates within diffuse fluids were higher for Piccard (1.2 × 10^4^ cells consumed ml^−1^ hr^−1^) than Von Damm (4.6 × 10^3^ cells consumed ml^−1^ hr^−1^). IGT results yielded a wide range of grazing rates; the averages of the non-zero grazing estimates were 1.1 × 10^4^ cells consumed ml^−1^ hr^−1^ at Piccard and 2.4 × 10^3^ cells consumed ml^−1^ hr^−1^ at Von Damm (Fig. 1d; Table 1). By incorporating biomass estimates of microbial prey, which relied on a carbon conversion factor of 86 fg C cell^−1^ [46], we determined the amount of carbon associated with the prokaryote population (based on microbial cell abundances) that may be taken up by the grazer community outside of the vent environment as 5.6 pg C ml^−1^ hr^−1^. Based on experiments run at ambient and *in situ* pressure, the rates of carbon consumption within the vent were 209 pg C ml^−1^ hr^−1^ and 980 pg C ml^−1^ hr^−1^, respectively (Table 4).

**Table 4:** Estimated amount of carbon consumed by the protistan grazer population.^a^

	**Average Clearance rate**	**Clearance rate (min/max)**	**Average amount of C consumed**	**Amount of C consumed (min/max)**
	**(pg C grazer** ^**−1**^**)**	**(pg C grazer** ^**−1**^**)**	**(μg C L** ^**−1**^ **day**^**−1**^**)**	**(μg C L** ^**−1**^ **day**^**−1**^**)**
Habitat type comparison				
Non-vent	0.10	0/0.1	0.13	0.05/0.26
Vent	1.20	0/3.9	14.27	0.24/35.68
Vent field comparison				
Piccard (vent only)	2.50	1.1/3.9	9.39	0.24/32.75
Von Damm (vent only)	0.50	0/1.4	24.04	12.4/35.68
Comparison of experimental approach				
Shipboard (vent only)	1.86	0.26/3.86	23.53	2.17/35.68
IGT (vent only)	0.49	0.02/1.14	5.02	0.24/12.4

a Based on the clearance and grazing rates (Tables 1, 2, S3; Fig. 1), assuming the amount of carbon represented by each prokaryotic cell is 86 fg C [31]. Calculations for all estimates are derived from equations listed in Table 2. Values reported below represent the average, minimum, and maximum pg of carbon consumed using clearance rate (mL grazer^−1^ hr^−1^) and grazing rate (cells consumed ml^−1^ hr^−1^). Data are grouped by the central comparisons relevant to the main text.

Grazing rates corresponded to the microbial cell abundances and temperature of fluid sampled at both Von Damm and Piccard (Fig. 2). The highest grazing rates (>1000 cells ml ^−1^ hr^−1^) were generally found at sites with higher concentrations of microeukaryotes (>300 cells ml ^−1^) and microbial prey cells (>1.0 × 10^5^ cells ml^−1^). Additionally, vent field and fluid temperature appeared to play a role in the trend between microbial prey concentration and protistan grazing rate (Fig. 2b). The highest protistan grazing rates at Piccard corresponded to the highest concentration of microbial prey and temperature maxima. By contrast, increasing temperatures at Von Damm (beyond 100°C) appeared to limit microbial prey concentration and subsequent grazing rate (Fig. 2b). Patterns observed between eukaryotic cell abundance, microbial prey abundance, temperature, and grazing rate were consistent, regardless of the pressure conditions of the incubation (Fig. 2). For four experiments in which grazing was deemed undetectable (negative slope), the temperature, vent fluid, and cells ml^−1^ did not show a predictable pattern. The unpredictability observed instead was attributed to the highly mixed, wafty, and ephemeral nature of the diffuse flow and seawater interface. Comparisons of grazing rate with other environmental parameters were not found to have a relationship (Fig. S7 and Table S6).

### Links to species composition

To investigate specific protistan taxonomic groups that may be linked to elevated grazing activity or hydrothermal vent habitat type, as well as how communities changed during the grazing experiments, we compared the community composition, derived from 18S rRNA gene sequence analysis performed across vent and non-vent samples and between the *in situ* microbial community collected by the HOG fluid sampler and Tf samples from the ambient grazing experiments (Fig. S8). Generally, the alveolate taxa, ciliates and dinoflagellates, outnumbered other recovered taxa in both species richness (ASV richness) and sequence number (comparative relative abundance). Second to the alveolates, hacrobia, rhizaria, and members of the stramenopile groups were consistently present across hydrothermal vents at the Mid-Cayman Rise (Fig. S8; also see [45, 46]). Since relative abundance of 18S rRNA gene amplicons is not representative of cell biomass and gene copies can vary significantly by species, we drew the majority of our observations from transformed data to minimize these artifacts [47, 48].

**Figure 3:**
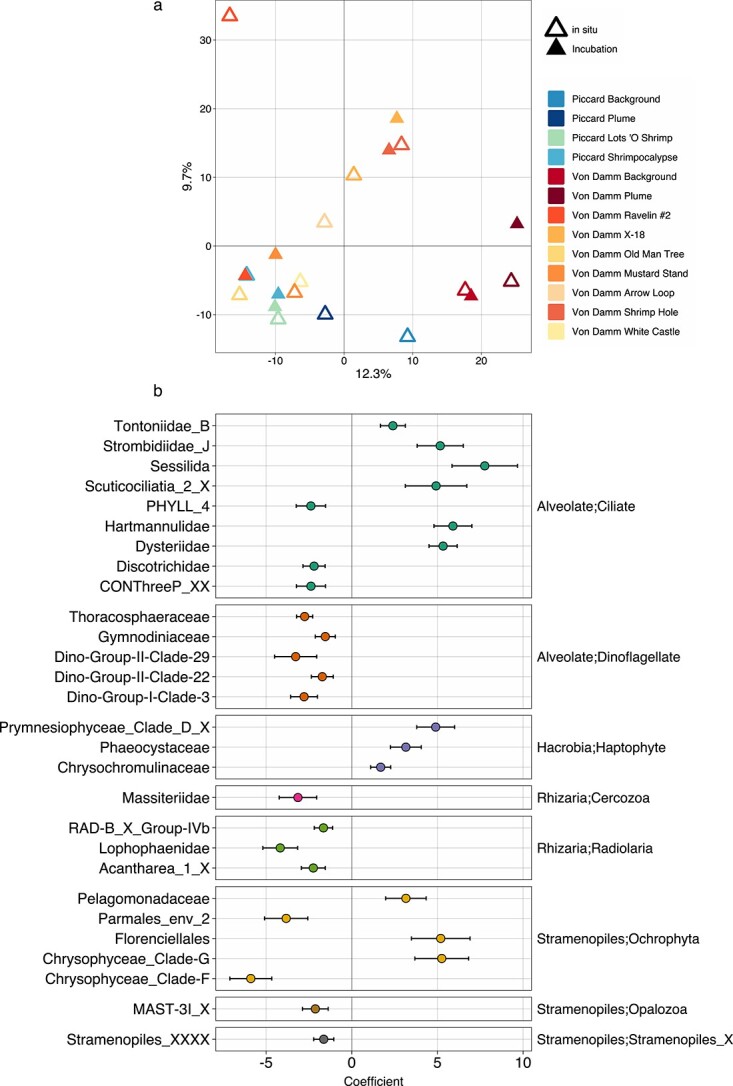
(a) Ordination analysis based on 18S rRNA gene amplicon sequencing. Samples include the *in situ* microbial eukaryotic community (open triangle symbols) and the Tf for grazing incubations conducted at ambient pressure (filled-in triangle symbols). No molecular samples were available from the IGT grazing experiments. Before PCA analysis, data were center-log ratio transformed. The x and y axes represent 12.3% and 9.7% of the variability among samples, respectively. Color designates each vent site, plume, or background sample and symbol differentiates the vent field. (b) Output from corncob analysis [33], which identified specific families that may be enriched within vent samples (positive coefficient) compared to non-vent samples (negative coefficient; includes background and plume).

Ordination analysis revealed that the community composition of protistan communities from diffuse fluid generally clustered with corresponding Tf grazing experiment samples (open versus shaded symbols in Fig. 3a). ASVs that appeared in both *in situ* samples and samples from grazing incubations were assumed to represent taxa contributing to grazing; of these ASVs, over 1500 were found to be shared at the Piccard and Von Damm sites and the majority were also cosmopolitan (found at vent and non-vent sites) (Table S7). Comparisons between *in situ* and grazing Tf samples at the ASV level revealed a higher occurrence of dinoflagellates, radiolaria, and opalozoa in non-vent experiments compared to vent-site experiments (Fig. S8). Overall, ciliates and dinoflagellates appeared to be the predominant protistan grazers in all experiments (Fig. S8).

Positive coefficients derived from corncob analyses of sample data demonstrated greater enrichment at the taxonomic family level of samples collected at vent sites than samples collected at non-vent sites (Fig. 3b). These results show that for major taxonomic groups, specific families are enriched within the vent samples; including families within the ciliates, haptophyta, and ochrophyta. For instance, many of the ciliate groups had positive coefficients, such as the *strombidiae* and *scuticociliates*, while most other families did not. Although most dinoflagellate families were not enriched at the vent sites compared to the non-vent samples, their prominence still suggested that they were a key player in the vent protistan community (Fig. 3b). Within the stramenopiles, *Pelagomonadales*, *Dictyochophyceae*, and Clade G of *Chrysophyceae* were the only families to show consistent enrichment at the vent sites.

## Discussion

**Figure 4:**
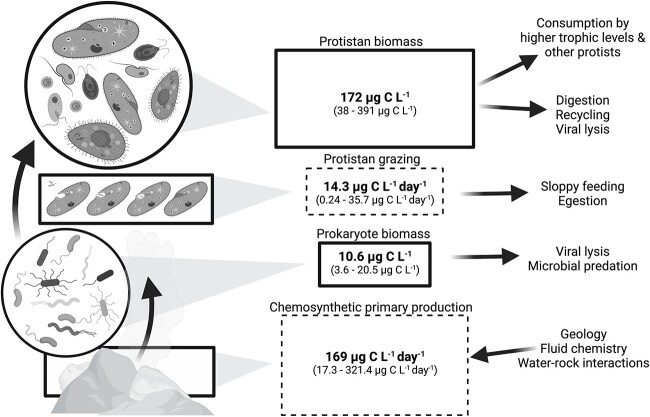
Schematic of the microbial food web at the Mid-Cayman Rise hydrothermal vent fields in terms of carbon. Rates are expressed as μg C L^−1^ day^−1^ (dashed line boxes) and biomass is represented by μg C L^−1^ (solid line boxes). Values are derived from experiments conducted with diffuse flow vent fluid and list the reported average (bolded), minimum, and maximum (parenthetical). Arrows show the net flow of carbon to higher trophic levels and unconstrained losses. In order to show results alongside primary production, we included the range of chemosynthetic primary production derived from McNichol et al. [33]. Eukaryote and prokaryote biomass was determined by multiplying carbon conversion factors by cell abundances from this study (see Table 2 for equations). Protistan grazing rate was calculated by converting predation rate into μg of carbon (see Tables 3 and 4). Image created with BioRender.com.

We quantified microbial eukaryotic cell concentrations and predation pressure across two deep-sea hydrothermal vent fields using grazing experiments conducted at both ambient (1 atmosphere) and *in situ* deep-sea pressures. Our study at the Mid-Cayman Rise offered the opportunity to compare two vent fields, Von Damm and Piccard, which are located close together but at separate depths and have distinct geochemistry. The subsurface fluid venting from Von Damm is largely influenced by ultramafic rock and is known to contain less dissolved sulfide and to have higher concentrations of methane than the fluid venting from Piccard (Table S1). The combination of higher pressure (deepest known hydrothermal vent at ~4900 m) and mafic rock at the Piccard vent fields causes a unique signature of venting fluid that is more acidic and enriched in dissolved hydrogen than fluid from other basalt-hosted systems [49]. Regardless of vent field or experimental approach, our results revealed that sites of active diffuse flow attract a higher diversity and abundance of microorganisms that leads to increased rates of protistan grazing. The resulting data add to only one other set of published values for hydrothermal vent protistan grazing rates [10] and provide previously unquantified ranges for vent-associated microbial eukaryote cell abundance and biomass. Our aim is to place these results in the larger context of how carbon is exchanged in the hydrothermal vent microbial food web (Fig. 4). Therefore, since the ability to conduct these experiments under *in situ* conditions is not commonplace, we incorporated results from both deep-sea and ambient pressure experiments to constrain protistan grazing at deep-sea hydrothermal vent food webs.

### The importance of determining deep-sea microbial interactions *in situ*

Differences between experiments conducted with vent fluid kept in IGTs versus collected with ROV *Jason* reflect the influence that depressurization likely has on deep-sea protistan survival and activity. Experiments conducted at 1 atmosphere underestimated grazing rates and cell abundances. Biological replicates from Ravelin #2 (Von Damm) and Shrimpocalypse (Piccard) allowed direct comparison of microeukaryote cell abundances between IGT and ambient experiments (Table 1), despite the difference in incubation volume. Eukaryotic cell abundances and carbon biomass at Ravelin #2 and Shrimpocalypse were consistently higher within IGT experiments, which we interpret as *in situ* pressure maintaining cell structure and integrity [17, 19]. Consistent with this observation, average eukaryotic cell abundances (Fig. 1a) and biomass (Tables 1 and 3) of the vent fields were slightly higher for the Von Damm than for the Piccard. Since the Piccard vent fluid collection process would experience a larger change in pressure, we speculated that this process contributed to differences in the downstream results.

Molecular analyses revealed the microbial eukaryotic community composition to be similar between the *in situ* vent fluid and final time point of each grazing experiment (Figs. S8 and 3a). This finding provides evidence that our shipboard experiments largely captured and retained microbial communities representative of the deep sea. Since the majority of ASVs shared between the *in situ* diffuse fluid samples and grazing incubations were found at both vent fields and present throughout the vent and non-vent habitats (Table S7), we hypothesized that collection and depressurization ahead of the shipboard incubations selected for protists that are more ubiquitous throughout the deep sea (cosmopolitan), rather than isolated to hydrothermal vent sites. Further, many of the selected protists may include barotolerant taxa, a trait that exists in many species, but is highly variable and species-specific [19, 50]. The microbial prey population in our experiments was assumed to be representative of the diffuse vent community. This assumption is derived from previous evidence that when used in shipboard experiments the prokaryotic community remains compositionally similar to *in situ* communities, while gene expression results show evidence that cells experience environmental stress [21].

Despite differences in vent fluid origin and pressure condition of the grazing experiments, a similar relationship between microbial cell abundance, diffuse vent fluid temperature, and protistan grazing rates was found (Fig. 2). This observation provides further evidence that trophic interactions among hydrothermal vent protists, bacteria, and archaea require continued study. Adding to the value of pursuing experiments conducted at ambient and *in situ* pressures, when results from this study were compared with those from a previous deep-sea vent protistan grazing study that used a different experimental approach [10], grazing rates and minimum-maximum values were found to be comparable (Fig. S9; also see Supplemental Information). The Mid-Cayman Rise was also an ideal location for this comparison, as we found the same trends in protistan cell abundances and grazing rates at both the Piccard and Von Damm vent fields. Together, with the aforementioned challenges in conducting these experiments, we elected to use results from both *in situ* and ambient pressure grazing experiments to constrain the predation pressure that protists exert on hydrothermal vent microbial prey.

### Trends in microeukaryotic cell biomass and grazing activity

Protistan top-down pressure varied at separate vent fields and within the same vent field (Fig. 1c; Table 1). Individual vent sites (1–10 s meters apart) are known to host highly diverse and distinct microbial communities between vent sites [6], which likely contributes to the observed range in grazing rates [51]. Similarly, other studies that measure protistan grazing and biomass often observe a range of values. In particular, a study that used a sampling device to conduct experiments *in situ* reported grazing rates ranging from 18.7 to 13 600 cells ml^−1^ hr^−1^  [52], which was comparable to our results of 24–17 200 cells ml^−1^ hr^-1^.

Microbial eukaryotic cell abundances were enriched within diffuse vent fluids (average of 230–620 cells ml^−1^); at minimum, the concentration of eukaryotic cells in diffuse fluids was more than 2-fold higher than the concentration of non-vent seawater (90–150 cell ml^−1^). This trend parallels observations of bacterial and archaeal abundances at hydrothermal vents [53] as well as patterns of protistan community diversity and species richness, confirming previous hypotheses regarding microeukaryotic vent populations [3, 6]. These findings demonstrate how active diffuse flow produces, attracts, and supports a greater biomass and diversity of deep-sea microorganisms. Outside the range of direct diffuse flow, plume and background samples had eukaryotic cell counts comparable to those previously recorded from mesopelagic depths, which ranged from 74 to 400 cells ml^−1^ [30, 52, 54, 55]. Furthermore, this work contributes to growing evidence that deep-sea vents supply a substantial amount of labile carbon to the deep sea [15, 56].

Biomass estimates revealed that deep-sea microeukaryotes make up 391 μg C L^−1^ (Table 3) at the diffuse vent fluid–seawater interface, which has the potential to supply a substantial carbon resource for other vent organisms (Fig. 4). In Pernice et al. [30], protistan biomass was found to decrease with depth, from 0.28 μg C L^−1^ at 200–450 m to 0.05 μg C L^−1^ at 1401–4000 m. These values are lower than what was found in the non-vent environment at the Mid-Cayman Rise (12.9 μg C L^−1^; Table 3), which may be explained by the proximity of the hydrothermal vent to the plume and deep seawater in this study compared to the meso- to bathypelagic environment sampled previously [30]. The amount of carbon represented by the hydrothermal vent protistan community is also significant as it demonstrates that protists can serve as a food resource to higher order consumers (Table 3) [15]. Studies of larger macrofauna at vent sites suggest that their diets include isotopically varied food sources [57], which include microbial eukaryotes [58].

Paired molecular analyses show that ciliates, dinoflagellates, hacrobia, and stramenopiles can make up a large proportion of the grazer community (Figs. S8 and 3b), which is consistent with previous work [6, 10, 11]. The higher biomass measured within vent fluids may be explained by the increase in larger eukaryotes, such as ciliates (Table 3; Figs. 3b, S2, and S8). While heterotrophic flagellates, many of which are stramenopiles, have been documented as key grazers throughout the deep sea and mesopelagic [30], the increase in prey availability and resources at vent sites can sustain larger ciliate cells. Thiscorroborates observed increases in biomass detected at vents compared to non-vent environments in this study and previously [52], where there was an increase in ciliates, relative to flagellates, at a biological hotspot in the deep sea (halocline).

Diffuse vent sites with the highest recorded temperature typically included the highest concentrations of microeukaryotic and microbial prey cells, and subsequent grazing rates (Fig. 2). The mixing of heated, end-member hydrothermal fluid and cold oxygenated seawater generates an increase in available oxidants and reductants for increased microbial metabolic activity that ultimately enhances chemosynthetic productivity at diffuse vent sites [59]. This is especially true at ultramafic sites, like Von Damm, where both subsurface abiotic and biotic carbon synthesis within mixing vent fluid contributes to a higher availability of labile carbon [15, 59, 60]. Thus, between Piccard and Von Damm, we originally expected the highest grazing rates to be at Von Damm; however, average grazing rates at Piccard were higher relative to Von Damm (Fig. 1c). We attributed this to a temperature limitation; the highest temperatures at Von Damm (peak of 121°C during collection; Table S1) appeared to limit cell abundances, causing a plateau (Fig. 2). Factors controlling protistan grazing pressure are often found to be temperature and the abundances of predators and prey, similar to what we found, but temperature limitation on grazing capacity has also been observed [61]. We emphasize the role that cell abundance (eukaryotic and prokaryotic) and temperature play in this study (Fig. 2), as other environmental parameters did not demonstrate as clear of a trend (Fig. S7, Table S6); however, we acknowledge that co-varying parameters or other cryptic processes likely contribute to microbial food web dynamics in the deep sea (Fig. 2).

### Summary and broader implications

One of the critical links between the hydrothermal vent chemosynthetic microbial community and all other trophic levels is the ecological role of microbial eukaryotic grazers. We applied novel technologies to estimate deep-sea hydrothermal vent protistan populations and biomass, to provide an improved and more constrained view of the microbial foundation of these deep-sea hydrothermal vent food webs. Our findings indicate that best practice is to conduct experiments under *in situ* conditions. However, considering the constraints associated with the required access to technology and necessary time and effort to conduct *in situ* incubations in the deep sea, we also show that results from experiments at ambient pressure still contribute meaningful observations, as long as the limitations are acknowledged.

The amount of carbon biomass stored within the prokaryotic and eukaryotic microbiota is substantial and can vary as a factor of hydrothermal vent geochemistry, community composition, and temperature. Using carbon as the currency, we illustrate the potential range of carbon biomass and trophic transfer, beginning with chemosynthetic primary production (values derived from [33], moving to consumption (by protists), and extending to unconstrained routes of carbon export, such as viral lysis, sloppy feeding, or digestion (Fig. 4). The unconstrained routes of carbon flux shown in Fig. 4 also highlight how the influence of the hydrothermal vent microbial food web can extend beyond the local vent region. For instance, the rising diffuse vent fluid with entrained seawater forms a hydrothermal vent plume, which can influence productivity in the rest of the water column [15, 56, 58].

Marine food webs are considered prone to significant restructuring as we anticipate future disruptive activities in our oceans. Anthropogenically driven exploitation of deep-sea resources, including at hydrothermal vent sites, is expected to negatively impact biodiversity and, consequently, ecosystem function and associated ecosystem services. Studies like this one, which assess the diversity and ecological contributions of microbial communities to carbon flux, are both necessary and timely.

## Supplementary Material

SupplementaryInformation_wrae004

## Data Availability

All necessary data products and code to reproduce results can be found at https://shu251.github.io/midcayman-rise-microeuk/. Raw sequence data are available through NCBI, SRA BioProject accession number PRJNA802868 and BCO-DMO doi:10.26008/1912/bco-dmo.914399.1.
