# Supplementary material for: Microbial eukaryotic predation pressure and biomass at deep-sea hydrothermal vents
Source: ISME J. 2024 Jan 13;18(1):wrae004. doi: 10.1093/ismejo/wrae004 (PMC10939315; doi:10.1093/ismejo/wrae004)
Supplement: SupplementaryInformation_wrae004 [file supplementaryinformation_wrae004.zip › SupplementaryInformation_Hu-et-al_ISMEJ.docx]

***Supplementary Information for***

**Microbial eukaryotic predation pressure and biomass at deep-sea hydrothermal vents**

Sarah K. Hu^1,2 *^, Rika E. Anderson^3^, Maria G. Pachiadaki^4^, Virginia P. Edgcomb^5^, Margrethe H. Serres^2^, Sean P. Sylva^2^, Christopher R. German^5^, Jeffrey S. Seewald^2^, Susan Q. Lang^5^, & Julie A. Huber^2^

1. Department of Oceanography, Texas A&M University, College Station, TX, USA
2. Department of Marine Chemistry and Geochemistry, Woods Hole Oceanographic Institution, Woods Hole, MA 02543, USA
3. Biology Department, Carleton College, Northfield, MN 55057, USA
4. Biology Department, Woods Hole Oceanographic Institution, Woods Hole, MA 02543, USA
5. Department of Geology & Geophysics, Woods Hole Oceanographic Institution, Woods Hole, MA 02543, USA
6. **Preparation of Fluorescently-labeled prey**

Monocultures of *Hydrogenovibrio* [Strain MBA27; [1]] were grown in Luria Broth Base media (LB broth; 5 g tryptone, 2.5 g yeast extract, 5 g NaCl in 500 ml of Milliq water, at pH 7.0 and autoclaved). To avoid clumping or biofilm formation, 70 ml of culture was grown in 250 ml flasks at 35ºC on a shaker plate (115 RPM) overnight. Using a modified version of an established protocol [2], bacteria were concentrated by centrifugation at 7,500 RPM for 30 minutes to form a pellet of cells, suspended liquid was decanted, and the centrifugation was repeated. The pellet was resuspended to Sea Salt Broth (SSB) by vortexing vigorously for 10 minutes. SSB (5X) was prepared by mixing and autoclaving: 98g NaCl, 16.5g Na_2_SO_4_, 1.5g KCl, 0.25g KBr, 0.1g H_3_BO_3_, 0.1g and 44g of MgCl_2_-6H_2_O into 1 liter of sterile Milliq water.

SSB rinse steps were repeated three times to remove away additional LB broth, by repeatedly resuspending cells in 10 mL of SSB. Resuspended and rinsed cells were vortexed vigorously for 10 minutes again and then 150 µl of DTAF (filter sterilized stock of 5-(4,6- dichlorotriazin-2-yl) aminofluorescein) was added while cells were incubated in a water bath (60°C) for 2 hours. During the 2 hour heat-kill incubation, cells were vortexed every 10-15 minutes to maintain suspension and reduce clumping. After the 2 hour incubation, cells were centrifuged at 7,500 RPM for 20 minutes to pellet the heat-killed and stained cells. Excess liquid was removed and replaced with 10 ml of SSB. This rinsing step was repeated three times to remove excess DTAF stain.

Finally, resuspended cells in separate tubes were combined, vortexed thoroughly and aliquot into 1 ml volumes before being frozen at -80°C. The concentration of this FLP stock was estimated by preparing slides from randomly selected cryovials and counting under epifluorescence microscopy.

1. **IGT Technical replication**

Only 1 IGT experiment was permitted per ROV Dive, therefore biological replicates for IGTs were not carried out simultaneously. Replication across separate ROV dives and comparisons with shipboard experiments was attempted for Ravelin #2, Old Man Tree, and Shrimpocalypse (see Table 1 and Table S1). However, some of the grazing experiments did not work due to leakage during the IGT fluid collection process or were not countable due to particulates forming in the fixed samples. Due to these challenges, we relied on the biological replicates between IGT and shipboard at Ravelin #2 and Shrimpocalypse (reported in main text, see Table 1, IGT7). Unfortunately, while the eukaryote cell abundance from T0 was countable, the rest of the fixed samples formed precipitates and were not countable. This is common in fixed fluid from hydrothermal vents due to the geochemistry of subsurface fluid interacting with fixative. Nonetheless, we elected to include the eukaryotic cell abundance, as it served as a biological replicate for the ambient pressure experiment from Shrimpocalypse (Table 1).

To augment our comparison between the IGT and shipboard experiments, we chose to re-count the IGT results as “technical replicates”. This served to ground truth our IGT experiments (denoted with “b” in Figure S10). Our results showed the technical replicates to be comparable (Figure S10). Together with the consistent patterns between the IGT- and shipboard-conducted experiments, we determined that for the purposes of constraining protistan grazing on microbial prey at hydrothermal vent sites, using both the IGT- and shipboard-based results is suitable. However, to be conservative in our interpretations we did not want to average across biological and technical replicates, thus these are kept separate in our analysis.

1. **Eukaryotic cell size and biomass**

Due to the cell shrinkage from fixation and depressurization from the deep sea, we determined that microscopic designations between micro (> 20µm) and nano (<20 µm) were not accurate [3, 4]. Assignment of the nano- vs. micro-size classes was inconsistent, as the effects of formaldehyde fixation and depressurization will not be consistent across all cell types. While results from micro- and nano-eukaryote counts are reported throughout the study, major interpretations and discussion points are based on the biovolume-derived carbon estimates. The nano-, micro-, and total eukaryotic cell counts are shown in Figure S2.

1. **Grazing experiment control samples**

Control bottles for grazing experiments serve to ensure the introduced FLP do no decrease due to other factors (Figure S6). For all shipboard experiments, controls were performed in duplicate (REP1 and REP2 in Figure S6a). IGT controls were conducted asynchronously from the IGT experiments. Due to the limited number of IGT controls, a technical replicate was counted for IGT control 0122 (see IGT-CTRL-0122-a and IGT-CTRL-0122-b in Figure S6b).

Variation between the replicates is acceptable, as the goal is to ensure that the FLPs remain stable over time. FLP counts that appear to deviate from the initial spiked FLP concentration (*e.g.,* Plume Von Damm, IGT-CTRL-0119-a) can be explained by insufficient mixing after the FLPs are introduced. Similarly, if the FLPs did not necessarily remain stable over time, but the replicates showed identical trends over time, this can also be attributed to sample-specific insufficient mixing (Shrimpocalypse, Piccard in Figure S6).

**References for Supplementary Information**

1. Trembath-Reichert E, Butterfield DA, Huber JA. Active subseafloor microbial communities from Mariana back-arc venting fluids share metabolic strategies across different thermal niches and taxa. *ISME J* 2019; **13**: 2264–2279.

2. Sherr BF, Sherr EB, Fallon RD. Use of monodispersed, fluorescently labeled bacteria to estimate in situ protozoan bacterivory. *Appl Environ Microbiol* 1987; **53**: 958–965.

3. Choi JW, Stoecker DK. Effects of fixation on cell volume of marine planktonic protozoa. *Appl Environ Microbiol* 1989; **55**: 1761–1765.

4. Edgcomb VP, Taylor C, Pachiadaki MG, Honjo S, Engstrom I, Yakimov M. Comparison of Niskin vs. in situ approaches for analysis of gene expression in deep Mediterranean Sea water samples. *Deep Sea Res Part II* 2016; **129**: 213–222.
