# Supplementary material for: Microbial eukaryotic predation pressure and biomass at deep-sea hydrothermal vents
Source: ISME J. 2024 Jan 13;18(1):wrae004. doi: 10.1093/ismejo/wrae004 (PMC10939315; doi:10.1093/ismejo/wrae004)
Supplement: SupplementaryInformation_wrae004 [file supplementaryinformation_wrae004.zip › SupplementaryInformation_wrae004.docx]

**Figure S1**. Images of sample collection and microeukaryotic cells from the Mid-Cayman Rise. ROV Jason sampling at **(a)** Lots O Shrimp (Piccard), **(b)** Ravelin #2 (Von Damm), and **(c)** Shrimpocalypse (Piccard). **(d)** ROV Jason manipulator arm holding the Isobaric Gas Tight (IGT) chamber. Image credit from ROV Jason: Woods Hole Oceanographic Institution and Jeffrey Seewald. Microeukaryotic cells captured with epifluorescence (DAPI filter) from FLP uptake experiments conducted in this study. **(e-j),** where emitted blue light is from DAPI stained cellular structures and emitted yellow is from the FLP. All images are derived from shipboard experiments and scale bars are located within each panel. **(e-g)** Eukaryotic cells from grazing experiments conducted at vent site X-18. The cell in panel **(e)** contained a consumed FLP cell. Imaged cells from **(h)** Mustard Stand, **(i)** Shrimpocalypse, and **(j)** the plume at Von Damm. Two FLP cells are imaged inside the eukaryotic cell in (j).

**Figure S2.** Eukaryotic cell counts from all grazing samples (x-axis is cells ml^-1^; log scale), where color designates the micro- and nano- size classes in addition to the total eukaryote cell counts (sum of the micro- and nano- counts). See Supplementary Information for additional information on the micro- and nano- size classes. Left panels represent cell counts from IGT experiments (*in situ* pressure) and right-hand panels originate from shipboard experiments (ambient pressure). Note the differences in the x-axis scale between the IGT and shipboard results. Top panels are derived from experiments conducted with Von Damm sample fluid and bottom panels show results from Piccard. Boxplots outline the first and third quartiles (25^th^ and 75^th^ percentiles) and the vertical line denotes the median. Whiskers extend from the top and bottom of the boxes at 1.5 * the inter-quartile range.

**Figure S3.** Estimated range and mean of protistan biomass based on the measured biovolume of imaged cells (Figure S1) and the carbon conversion factor that incorporates a diverse group of protists, excluding diatoms [25] (also see Tables 2 and S4).

**Figure S4.** Results from conducted grazing experiments at **(a)** ambient pressure and **(b)** deep-sea pressure, where experiment time is along the x-axis and the number of FLP eukaryotic cell^-1^ detected is noted at the y-axis. The number indicated at the top each panel indicates the calculated slope of the line. For values that are negative, this is noted as undetected and reflected as zero in the main results. For IGT samples (b), the final time point at T40 (T3) was removed, as the small incubation chamber size likely caused an anomalous uptick in protistan death [10] (Figure S5).

**Figure S5**. Total eukaryotic cells ml^-1^ at each time point for IGT (left panels) and shipboard (right panels) experiments. Top panels are derived from Von Damm experiments and bottom panels are from Piccard. Note that the IGT experiments appear to have a bottle effect (smaller volume), so the final time point (T3, from approximately 40 minutes) was removed for downstream analyses.

**Figure S6.** FLP control experiments from **(a)** experiments run at ambient pressure and **(b)** *in situ* pressure. (a) Each panel represents a shipboard experiments, where control experiments were typically conducted in duplicate. Replicate FLP counts are denoted by symbols (REP1 vs. REP2) and separate lines. Missing data points occurred when preserved fluid was uncountable. (b) For the IGT experiments, plume and background seawater was used to mimic the control experiment. Due to the nature of IGT sampling, control samples could not be run at the same time as the experimental, therefore three total control experiments were conducted, 0119, 0122, and 0130. Counts for the experiment 0122 were done twice (a versus b) for technical replication. Grey shading in all panels shows 15% above and below the starting time point, T0. Ideally, FLP concentrations in control experiments will remain stable over time. For most experiments shown here, FLP concentration varied little with respect to time. FLP counts that deviated away from a straight line overtime can be explained by insufficient mixing of the control bottles during the experiment. For example, the counts for the plume experiment at Von Damm, remain stable over time, except for T0 for REP2, which appears higher (a). Additionally, no other anomalous trends were observed in eukaryotic cell counts over time (Figures S2 and S5). See Supplementary Information for added context.

**Figure S7.** All environmental parameters (y-axes) vs. protistan grazing rate (cells consumed ml^-1^ hr^-1^ (x-axis). Each panel represents a separate environmental parameter (Table S1). Values above each panel are the r^2^ values derived from a linear regression comparing grazing rate and each parameter (Table S6). Eukaryotic and prokaryotic cells ml^-1^ and temperature (°C) appeared to have a relationship with grazing rate and are investigated further in the main text. While this statistical approach is useful in exploring trends between vent geochemistry and protistan grazing activity, the limitation in the number of experiments and ability to compare across pressure conditions made statistical power somewhat limited (discussed in the main text and Supplementary Information).

**Figure S8.** Relative sequence abundance (y-axis) across the Mid-Cayman Rise *in situ* samples collected via the HOG fluid sampler and samples taken after the Tf (T40) time point for each grazing experiment conducted shipboard. Colors designate major taxonomic groups. If a grazing incubation sample (from Tf) is paired with the *in situ* community sample, they are positions next to one another. Samples without a paired grazing experiment are shown by themselves. Names along the x-axis report the vent field, environment type (vent, plume, background, or incubation), year of study, and (if applicable) the vent site name. A more detailed analysis of overall microbial eukaryotic diversity and distribution has previously been published [44]. Additional information on shared ASVs is reported in Table S7.

**Figure S9.** Comparison of grazing rates across three hydrothermal vent fields: Gorda Ridge [46], Von Damm, and Piccard (this study). Rates are expressed in log scale as the number of cells consumed by protistan predators ml^-1^ hr^-1^ (Grazing rate). Symbol color denotes vent field, filled in circles are derived from shipboard experiments or samples, while circle outlines represent results from IGT experiments.

**Figure S10.** Technical replicates for Old Man Tree (top panel) and Ravelin #2 (bottom panels). Since biological replicates were difficult to obtain for the IGT experiments, two halves of a filter were counted separately as technical replicates. On the right side are the results in this study and the left side reports results from “IGTxb” technical replicates. Together with grazing rates that were in range of another study (Figure S9), we can provide additional support in our findings (Supplementary Information).

**Table S1.** Metadata for all samples depth (meters), maximum temperature (°C) at time of collection, pH, estimated percent seawater in diffuse fluids (%), pH, magnesium (Mg mmol/L or mM), dissolved hydrogen (H2 μmol/L or μM), total dissolved hydrogen sulphide (H2S mmol/L or mM), and methane (CH4 μmol/L or μM), concentration of microorganisms (cells/ml), location, and fluid origin type.

**Table S2.** Complete experiment details for all shipboard and IGT grazing assays, including ROV Jason number, time points, number of replicates, controls, and volumes. Table also reports major results from cell counts, grazing rate, clearance rate, and percent bacterial turnover percentage.

**Table S3.** List of all variables and calculations used in this study and the variables corresponding to the R code. Equations, units, and references are also listed in Table 2, while this table reports additional parameters shown in the R code.

**Table S4.** Calculation of microbial eukaryotic biovolume from Hillebrand et al. [32] and estimate of carbon content cell^-1^ based on Menden-Deuer & Lessard 2000 [32]. Carbon conversion factors included the dinoflagellate-only based value and the mixed protistan community without diatoms. Both equations are listed in Table S3, but the main text of this study uses the field standard, mixed protistan community without diatom value.

**Table S5.** Estimates of protistan biomass in pg C cell^-1^ derived from biovolume and the carbon conversion factor that relied on the mixed protistan community without diatoms.

**Table S6.** Results from linear regression, comparing grazing rate variables, grazing rate (cells consumed ml^-1^ hr^-1^), clearance rate (mL grazer^-1^ hr^-1^), Specific grazing rate (Prokaryotes grazer^-1^ hr^-1^), and bacteria turnover (% removed day^-1^), to environmental and biological parameters measured, such as temperature, cell abundances, pH, and more. High r-squared values were typically associated with eukaryote cell abundances and are therefore further explored in the main text (Figure 2). Due to the limited number of biological replicates in this study, we have interpreted these statistical results conservatively; additional experimentation will be needed to prove the observed relationships.

**Table S7.** List of Amplicon Sequence Variants (ASVs) feature IDs that are shared between the *in situ* microbial community at the Mid-Cayman Rise and the shipboard grazing experiments. Columns indicate if the ASVs was distributed across both Piccard and Von Damm vent fields (Distribution by Site) and if it was considered Vent only or Resident (found only at hydrothermal vents) or cosmopolitan (found throughout vent, plume, and or background samples). Taxon lists the full taxonomic identity of the ASV based on taxonomic classification.
