# Supplementary material for: Microbial eukaryotic predation pressure and biomass at deep-sea hydrothermal vents
Source: ISME J. 2024 Jan 13;18(1):wrae004. doi: 10.1093/ismejo/wrae004 (PMC10939315; doi:10.1093/ismejo/wrae004)
Supplement: SupplementaryInformation_wrae004 [file supplementaryinformation_wrae004.zip › TableS1_wrae004.pdf]

Table S1.

FIELD	VENT	SAMPLETYPE	FLUID_ORIGIN	Depth (m)	Temperature (°C)	pH	Percent Seawater	Mg mmol/L (mM)	H2 µmol/L (µM)	H2S mmol/L (mM)	CH4 µmol/L (µM)	Microbial concentration (cells/mL)	Lat, Long	Type
Piccard	BSW	Background	CTD005	4776	4.5	nd	100	53.7	nd	nd	nd	1.2E+04	18.547980, -81.718180	CTD
Piccard	Plume	Plume	CTD004	4944	4.5	nd	100	nd	nd	nd	nd	5.1E+04	18.546767, -81.718200	CTD
Piccard	LotsOShrimp	Vent	J21242HOG14	4967	19.0	6.32	99.6	53.5	1.33	nd	11.9	5.4E+04	18.546789, -81.718356	HOG
Piccard	Shrimpocalypse	Vent	J21240HOG14	4945	85.0	5.11	81.7	43.9	0	nd	27.5	2.4E+05	18.546674, -81.717806	HOG
Piccard	LotsOShrimp	Vent	J21241HOG14	4967	36.0	5.92	94.9	51	22700	nd	11.5	5.4E+04	18.546789, -81.718356	HOG
Von Damm	BSW	Background	CTD002	2400	4.2	nd	100	nd	nd	nd	nd	3.5E+04	18.374183, -81.781533	CTD
Von Damm	Plume	Plume	CTD003	1979	4.2	nd	100	nd	nd	nd	nd	1.6E+04	18.377600, -81.799317	CTD
Von Damm	ArrowLoop	Vent	J21243HOG18	2309	137.0	5.69	33.8	18.1	11500	1.74	1893.7	1.0E+04	18.376659, -81.797986	HOG
Von Damm	WhiteCastle	Vent	J21235HOG12	2307	108.0	5.49	16.6	8.9	14500	1.96	2251.9	nd	18.377005, -81.798088	HOG
Von Damm	MustardStand	Vent	J21243HOG14	2374	108.0	5.63	36.2	19.4	9800	1.79	1820.0	5.7E+04	18.375130, -81.797488	HOG
Von Damm	Rav2	Vent	J21238HOG14	2389.6	94.0	5.81	33.4	18	10200	1.37	1893.7	nd	18.375112, -81.797180	HOG
Von Damm	OldManTree	Vent	J21238HOG20	2375.8	121.6	5.69	25.6	13.7	11600	1.77	1985.8	nd	18.375069, -81.797678	HOG
Von Damm	ShrimpHole	Vent	J21244HOG18	2376	21.0	7.72	96.4	51.8	5.52744	nd	218.0	nd	18.374893, -81.797441	HOG
Von Damm	X18	Vent	J21235HOG20	2377	48.0	6.99	52	28	nd	2.11	1310.2	1.1E+05	18.374810, -81.797411	HOG
Von Damm	Bartizan	Vent	J21244HOG12	2307	129.0	5.8	42.2	22.7	9400	1.6	1617.3	1.6E+04	18.798096, -81.377907	HOG
Von Damm	Rav2	Vent	J21244HOG20	2388.9	98.2	5.81	33.4	18	10200	1.37	1893.7	nd	18.375254, -81.797176	HOG

nd indicates no data available
