# Supplementary material for: Microbial eukaryotic predation pressure and biomass at deep-sea hydrothermal vents
Source: ISME J. 2024 Jan 13;18(1):wrae004. doi: 10.1093/ismejo/wrae004 (PMC10939315; doi:10.1093/ismejo/wrae004)
Supplement: SupplementaryInformation_wrae004 [file supplementaryinformation_wrae004.zip › TableS2_wrae004.pdf]

Table S2.

Vent Field	Site Name	ROV Jason dive number of CTD cast	Shipboard bag or IGT experiment	Experiment condition	Time points	Number of replicates (treatment)	Volume of treatment (L)	Number of replicates (control)	Volume of control (L)	Average eukaryote cells ml^{-1}	Eukaryote cell ml^{-1} (min / max)	Standard mean error eukaryote cells ml^{-1}	Average prokaryote cells ml^{-1}	Prokaryote cell ml^{-1} (min / max)	Standard mean error prokaryote cells ml^{-1}	FLP ml^{-1}	Grazing rate day^{-1}	Slope (*m*)	FLPs $grazer^{-1}$ min^{-1}	Clearance rate mL $grazer^{-1}$ hr^{-1}	Bacteria turnover (% removed prokaryotes day^{-1})
Piccard	Lots 'O Shrimp	J2-1241	LV24	Ambient	0, 15, 20, 40	3	1.5	2	0.2	2.31E+02	230.9 / 230.9	--	5.39E+04	26240 / 90260	13727	617	0	-0.0076	0.0000	0.000000	0.00
Piccard	Plume	CTD004	Niskin 10	Ambient	0, 10, 15, 20, 40	3	2	2	0.5	7.93E+01	55.98 / 112	17	5.14E+04	46810 / 56050	4618	29650	13	0.0054	0.0054	0.000011	2.07
Piccard	Shrimpocalypse	J2-1240	LV13	Ambient	0, 10, 14, 22, 42	3	1.5	2	0.2	4.55E+02	454.8 / 454.8	--	2.39E+05	108700 / 322000	65793	17003	317	0.0157	0.0157	0.000055	60.42
Von Damm	Background	CTD002	Niskins 8-10	Ambient	0, 10, 15, 20, 40	3	2	2	0.5	9.18E+01	69.97 / 113.7	22	3.79E+04	18470 / 56470	8608	4862	33	0.0030	0.0030	0.000037	8.05
Von Damm	Mustard Stand	J2-1243	LV17	Ambient	0, 10, 20, 40	2	1.5	2	0.2	2.60E+02	230.9 / 288.6	29	5.67E+04	42400 / 70950	14274	6601	0	-0.0054	0.0000	0.000000	0.00
Von Damm	Plume	CTD001	Niskin 2	Ambient	0, 10, 15, 25, 57	3	2	2	1	1.58E+02	55.98 / 284.4	67	1.65E+04	13850 / 19100	2624	34242	4	0.0053	0.0053	0.000009	3.50
Von Damm	Ravelin #2	J2-1238	LV13a	Ambient	0, 10, 15, 21, 40	3	1.5	2	0.5	4.09E+02	335.9 / 482.8	73	7.11E+04	--	--	51891	7	0.0035	0.0035	0.000004	3.94
Von Damm	Shrimp Hole	J2-1244	LV13	Ambient	0, 10, 15, 20, 40	2	1.5	2	0.2	3.86E+02	377.8 / 393.6	8	4.20E+04	38830 / 45130	3149	113354	0	-0.0020	0.0000	0.000000	0.00
Von Damm	X-18	J2-1235	LV23 & Bio5	Ambient	0, 15, 20, 40	2	1.5	2	0.5	3.15E+02	209.9 / 419.8	105	1.11E+05	108500 / 114400	2974	3149	89	0.0017	0.0017	0.000033	25.12
Piccard	Shrimpocalypse	J2-1240	IGT3	*in situ*	0, 15, 25	1	0.15	--	--	3.85E+02	384.8 / 384.8	--	2.39E+05	108700 / 322000	65793	5353	1078	0.0168	0.0168	0.000188	173.87
Von Damm	Old Man Tree	J2-1238	IGT4	*in situ*	0, 7, 25	1	0.15	--	--	3.50E+02	349.9 / 349.9	--	7.11E+04	--	--	5353	72	0.0038	0.0038	0.000042	35.43
Von Damm	Ravelin #2	J2-1244	IGT4	*in situ*	0, 12, 20	1	0.15	--	--	9.45E+02	944.6 / 944.6	--	7.11E+04	--	--	5353	403	0.0211	0.0211	0.000236	535.24
Von Damm	Ravelin #2	J2-1244	IGT5	*in situ*	0, 35, 40	1	0.15	--	--	6.30E+02	629.7 / 629.7	--	7.11E+04	--	--	5353	0	-0.0029	0.0000	0.000000	0.00
Piccard	Shrimpocalypse	J2-1240	IGT7*	*in situ*	--	--	--	--	--	5.25E+02	524.8 / 524.8	--	2.39E+05	108700 / 322000	65793	--	--	--	--	--	--

If slope was negative, it is shown as a zero above

*IGT7 from Shrimpocalypse was used for counting eukaryotic cells ml-1, but the grazing treatments were not countable.
