# Supplementary material for: Microbial eukaryotic predation pressure and biomass at deep-sea hydrothermal vents
Source: ISME J. 2024 Jan 13;18(1):wrae004. doi: 10.1093/ismejo/wrae004 (PMC10939315; doi:10.1093/ismejo/wrae004)
Supplement: SupplementaryInformation_wrae004 [file supplementaryinformation_wrae004.zip › TableS3_wrae004.pdf]

Table S3.

Grazing calculations

Category	VAR (grazing.qmd)	Equation or value	Units	Description	Citation
Cell concentration for prokaryotes	PROK_ml	(Total cells counted * area of counting grid)/(Volume of sample * volume of grid * dilution factor)	cells/ml	Cells per ml for bacteria and archaea. If cell count was 'uncountable', the average of all cell counts was used (7.11x 10^4)	
Cell concentration for eukaryotes	EUK_ml	(Total cells counted * area of counting grid)/(Volume of sample * volume of grid * dilution factor)	cells/ml	Cells per ml for eukaryotes	
FLP per ingested cell	FLPperEuk	Derived from microscopy counts	FLP/cell	Concentration of FLP inoculated into each experiment	
Time point for sample	Minutes	Minutes for T0	minutes	Time points in mintes	
Slope of best fit line	FLPs grazer-1 min-1	lm_out <- lm(FLPperEuk ~ Minutes, data = INPUT)	FLPs grazer-1 min-1	Slope of best fit line, derived in R	Sherr and Sherr, 1993
FLP concentration at T0	FLP_ml	(Total cells counted * area of counting grid)/(Volume of sample * volume of grid * dilution factor)	cells/ml	FLP concentration after inoculation	Unrein et al. 2007
Clearance rate	CLEARANCE_RATE_ml	(FLPs grazer-1 min-1) * (60 minutes) / (FLP_ml)	nL or mL grazer-1 hr-1	Volume that a protistan grazer can clear within an hour	Unrein et al. 2007
Specific grazing rate per hr	Prokaryotes grazer-1 hr-1	Clearance rate_ml * PROK_ml	prokaryotes grazer-1 hr-1	Numer of prokaryotes that a protistan grazer can consume in an hour	Unrein et al. 2007
Grazing rate per day	GRAZE_RATE_DAY	(Specific grazing rate per hr) * 24 hours	prokaryotes grazer-1 day-1	Numer of prokaryotes that a protistan grazer can consume in a day	Unrein et al. 2007
Grazing effect per hour	GRAZING_EFFECT_hr	(Specific grazing rate per hr) * EUK_ml	prokaryotes ml-1 hr-1	Number of prokaryotes per ml that the protistan grazer population can consume in an hour	Unrein et al. 2007
Cells consumed per ml per day	GRAZING_EFFECT_day	(Specific grazing rate per hr) * 24 hours * EUK_ml	prokaryotes ml-1 day-1	Number of prokaryotes per ml that the protistan grazer population can consume in a day	Unrein et al. 2007
Turnover percentage	Bacteria turnover % per day	100* (GRAZING_EFFECT_day / PROK_ml)	% removed prokaryotes day-1	Percent of the prokaryotic population that the protistan grazers consume in a day	Unrein et al. 2007

Determination of cell biomass

Category	VAR (grazing.qmd)	Equation or value	Units	Description	Citation
Biovolume	BIOVOLUME	pi / (6 * d^2 * h)	um^3	Assumes cell is a prolate spherical shape; Estimate biovolume by using d and h from microscopy	Hillebrand et al. 1999
Cell biomass (dinos only)	pgC_cell_MD_dino	0.760 x Vol ^0.819	pg C per cell	calculate C per cell	Menden-Duer & Lessard 2000
Cell biomass (non-diatoms)	pgC_cell_MD_nodiatom	0.216 x Vol ^0.939	pg C per cell	estimate to calculate C per cell	Menden-Duer & Lessard 2000
C conversion nano	c_n	2.6 pg C per cell	pg C per cell	0.183 pg C um^3; Volume = (4/3)*pi*r^3 = 2585 fg C per cell; convert	Caron et al. 1995
C conversion micro	c_m	138 pg C per cell	pg C per cell	Microplankton specific carbon conversion rate	Caron et al. 1995
C conversion bacteria	bac_carbon_Mor_fg	86 fg C per cell	fg C per cell	(Range in Trembath-Reichert et al. 2021 was reported as 21-443 fg	Morono et al. 2011
