# Supplementary material for: Microbial eukaryotic predation pressure and biomass at deep-sea hydrothermal vents
Source: ISME J. 2024 Jan 13;18(1):wrae004. doi: 10.1093/ismejo/wrae004 (PMC10939315; doi:10.1093/ismejo/wrae004)
Supplement: SupplementaryInformation_wrae004 [file supplementaryinformation_wrae004.zip › TableS4_wrae004.pdf]

Table S4.

Experiment type	Environment type	Height *(h)*	Depth *(d)*	Biovolume (μm^3)
IGT	vent	89.582	10	4690.50
IGT	vent	30.077	25.764	10453.45
IGT	vent	29.776	25.852	10419.65
IGT	vent	17.255	9.986	900.94
IGT	vent	10.991	4	92.08
IGT	vent	10.282	4.136	92.10
			**Average IGT**	**4441.45**
Shipboard	vent	41.153	21	9502.52
Shipboard	vent	36.164	3	170.42
Shipboard	vent	14.333	2	30.02
Shipboard	vent	10.069	5	131.80
Shipboard	vent	9.218	3.12	46.98
			**Average shipboard**	**1976.35**
			**Average vent-only**	**3208.90**
Shipboard	Background	16.206	14.924	1889.93
Shipboard	Background	14.595	8.036	493.49
Shipboard	Background	12.48	8.982	527.18
Shipboard	Background	7	7	179.59
			**Average background**	**772.55**

Cellular carbon content pg C $cell^{-1}$ (dinoflagellates only)	Cellular carbon content pg C $cell^{-1}$ (without diatoms)*
771.87	604.96
1487.95	1283.92
1484.01	1280.02
199.85	128.50
30.86	15.09
30.87	15.10
**667.57**	**554.60**
1376.14	1173.94
51.10	26.91
12.33	5.27
41.40	21.14
17.79	8.02
**299.75**	**247.05**
**483.66**	**400.83**
366.62	257.65
122.07	73.02
128.85	77.69
53.34	28.26
**167.72**	**109.16**
