# Supplementary material for: Microbial eukaryotic predation pressure and biomass at deep-sea hydrothermal vents
Source: ISME J. 2024 Jan 13;18(1):wrae004. doi: 10.1093/ismejo/wrae004 (PMC10939315; doi:10.1093/ismejo/wrae004)
Supplement: SupplementaryInformation_wrae004 [file supplementaryinformation_wrae004.zip › TableS5_wrae004.pdf]

Table S5.

Vent field	Experiment Condition	Sample type	Average cell carbon content (pg C ml^{-1})	Maximum cell carbon content (pg C ml^{-1})	Minimum cell carbon content (pg C ml^{-1})
Piccard	Ambient	Non-vent	8.7	8.7	8.7
Piccard	Ambient	Vent	142.2	188.7	95.8
Piccard	*in situ*	Vent	188.7	217.7	159.6
VD	Ambient	Non-vent	17.2	17.2	17.2
VD	Ambient	Vent	121.3	169.8	38.1
VD	*in situ*	Vent	237.0	391.8	145.1
