# Supplementary material for: Microbial eukaryotic predation pressure and biomass at deep-sea hydrothermal vents
Source: ISME J. 2024 Jan 13;18(1):wrae004. doi: 10.1093/ismejo/wrae004 (PMC10939315; doi:10.1093/ismejo/wrae004)
Supplement: SupplementaryInformation_wrae004 [file supplementaryinformation_wrae004.zip › TableS6_wrae004.pdf]

Table S6

Grazing rate or factor	Parameter	r-squared value	adjusted r-squared value
Grazing effect (hr^{-1})	Prokaryote cell count	0.445	-0.480
Grazing effect (hr^{-1})	Eukaryote cell count	1.000	NA
Grazing effect (hr^{-1})	Temperature	0.839	-0.285
Grazing effect (hr^{-1})	Depth (m)	0.839	-0.285
Grazing effect (hr^{-1})	pH	0.379	-0.552
Grazing effect (hr^{-1})	Percentage seawater	0.451	-0.648
Grazing effect (hr^{-1})	Mg	0.379	-0.552
Grazing effect (hr^{-1})	H2	0.524	0.238
Grazing effect (hr^{-1})	CH4	0.379	-0.552
Grazing effect (hr^{-1})	Prokaryote:Eukaryote cell count	1.000	NA
Clearance rate (ml)	Prokaryote cell count	0.296	-0.877
Clearance rate (ml)	Eukaryote cell count	1.000	NA
Clearance rate (ml)	Temperature	0.844	-0.249
Clearance rate (ml)	Depth (m)	0.844	-0.249
Clearance rate (ml)	pH	0.206	-0.985
Clearance rate (ml)	Percentage seawater	0.252	-1.245
Clearance rate (ml)	Mg	0.206	-0.985
Clearance rate (ml)	H2	0.357	-0.029
Clearance rate (ml)	CH4	0.206	-0.985
Clearance rate (ml)	Prokaryote:Eukaryote cell count	1.000	NA
Specific grazing rate (hr^{-1})	Prokaryote cell count	0.615	-0.025
Specific grazing rate (hr^{-1})	Eukaryote cell count	1.000	NA
Specific grazing rate (hr^{-1})	Temperature	0.707	-1.345
Specific grazing rate (hr^{-1})	Depth (m)	0.707	-1.345
Specific grazing rate (hr^{-1})	pH	0.536	-0.159
Specific grazing rate (hr^{-1})	Percentage seawater	0.576	-0.272
Specific grazing rate (hr^{-1})	Mg	0.536	-0.159
Specific grazing rate (hr^{-1})	H2	0.623	0.397
Specific grazing rate (hr^{-1})	CH4	0.536	-0.159
Specific grazing rate (hr^{-1})	Prokaryote:Eukaryote cell count	1.000	NA
Specific grazing rate (hr^{-1})	Prokaryote cell count	0.241	-1.025
Specific grazing rate (hr^{-1})	Eukaryote cell count	1.000	NA
Specific grazing rate (hr^{-1})	Temperature	0.973	0.788
Specific grazing rate (hr^{-1})	Depth (m)	0.973	0.788
Specific grazing rate (hr^{-1})	pH	0.285	-0.788
Specific grazing rate (hr^{-1})	Percentage seawater	0.332	-1.003
Specific grazing rate (hr^{-1})	Mg	0.285	-0.788
Specific grazing rate (hr^{-1})	H2	0.390	0.024
Specific grazing rate (hr^{-1})	CH4	0.285	-0.788
Specific grazing rate (hr^{-1})	Prokaryote:Eukaryote cell count	1.000	NA
