# Supplementary figures and images for: Microbial eukaryotic predation pressure and biomass at deep-sea hydrothermal vents
Source: ISME J. 2024 Jan 13;18(1):wrae004. doi: 10.1093/ismejo/wrae004 (PMC10939315; doi:10.1093/ismejo/wrae004)

Figure S1.

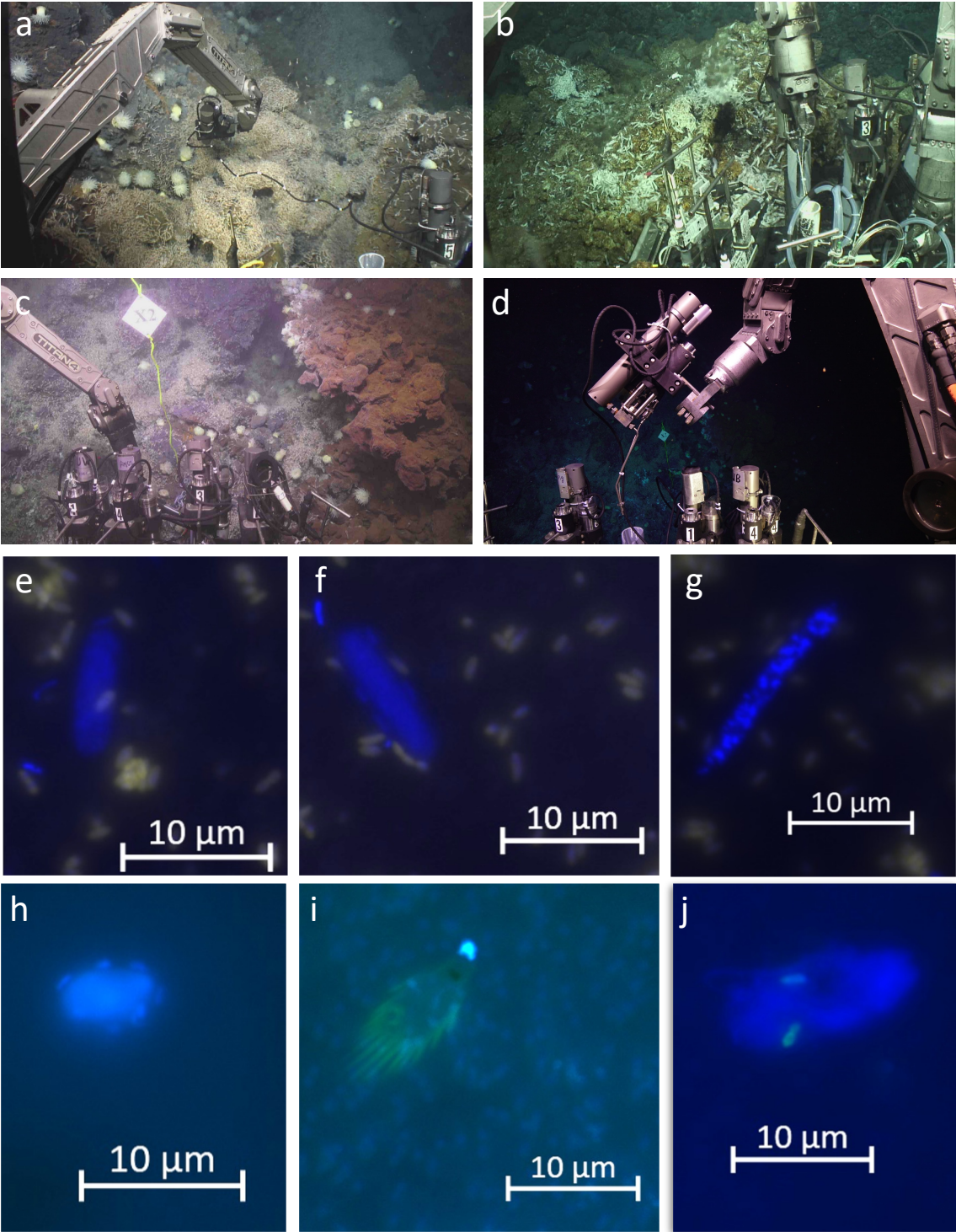

Supplement: SupplementaryInformation_wrae004 [file supplementaryinformation_wrae004.zip › figS1_new_wrae004.pdf]

Figure S10.

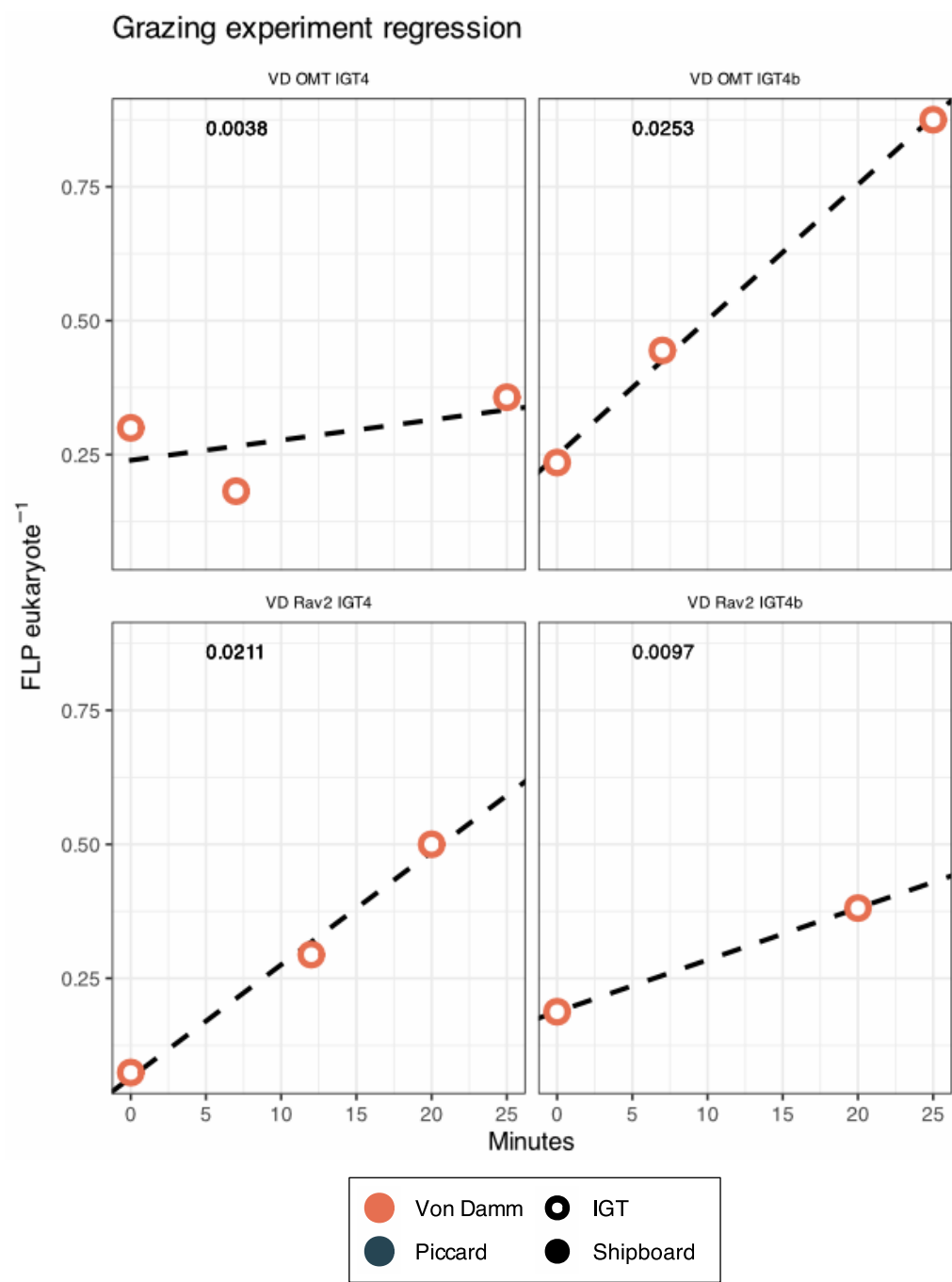

Supplement: SupplementaryInformation_wrae004 [file supplementaryinformation_wrae004.zip › figS10_new_wrae004.pdf]

Figure S2.

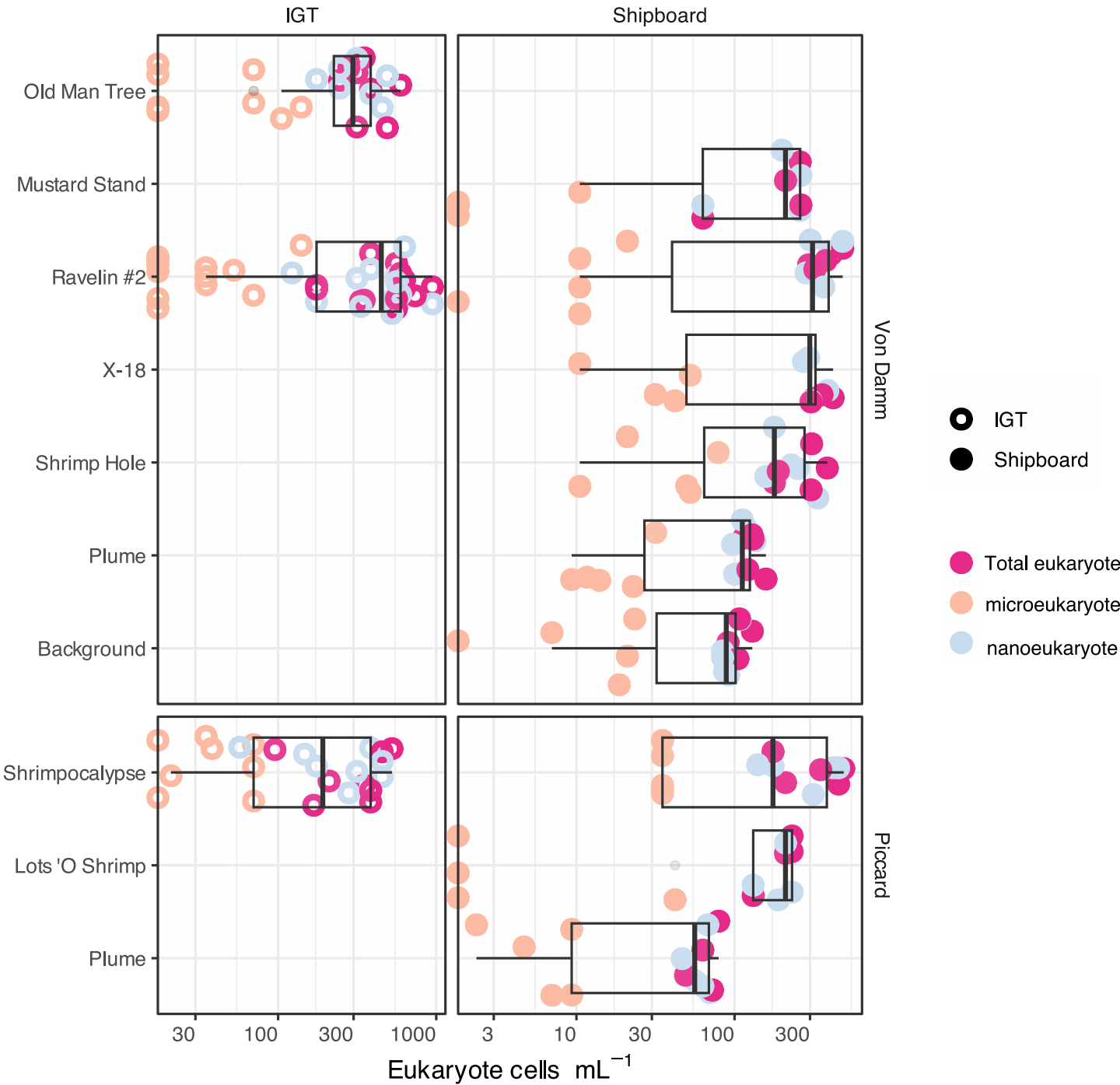

Supplement: SupplementaryInformation_wrae004 [file supplementaryinformation_wrae004.zip › figS2_new_wrae004.pdf]

Figure S3.

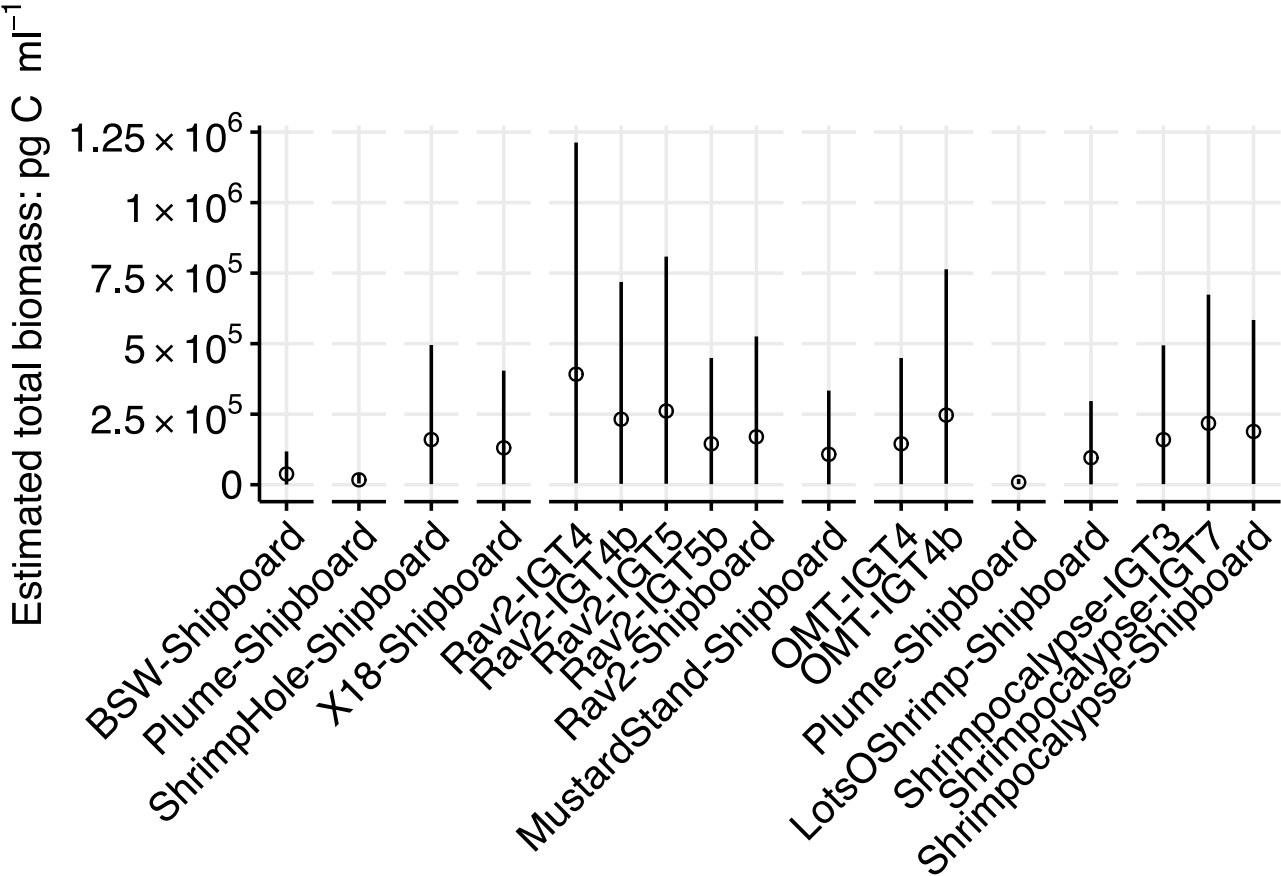

Supplement: SupplementaryInformation_wrae004 [file supplementaryinformation_wrae004.zip › figS3_new_wrae004.pdf]

Figure S4.

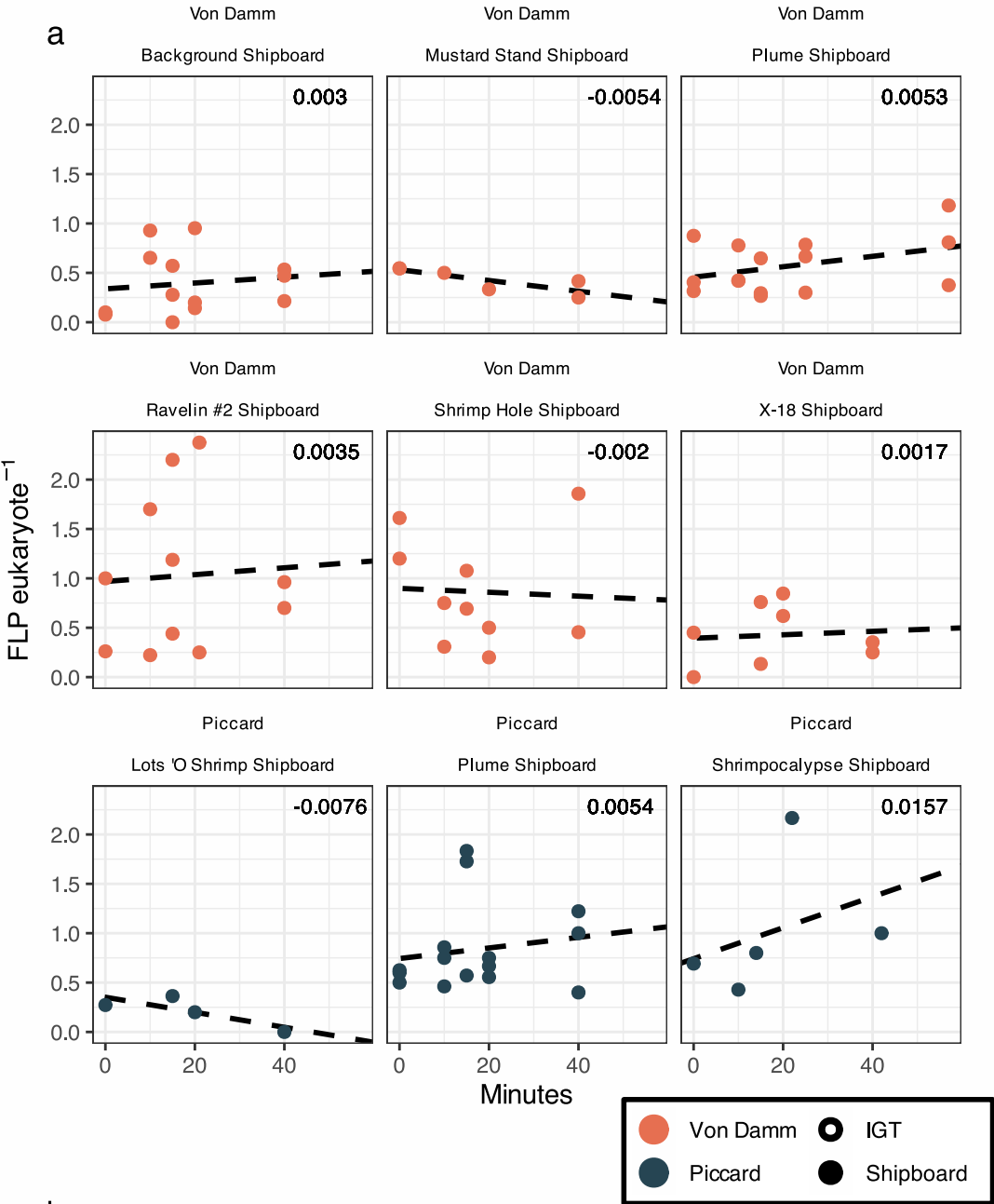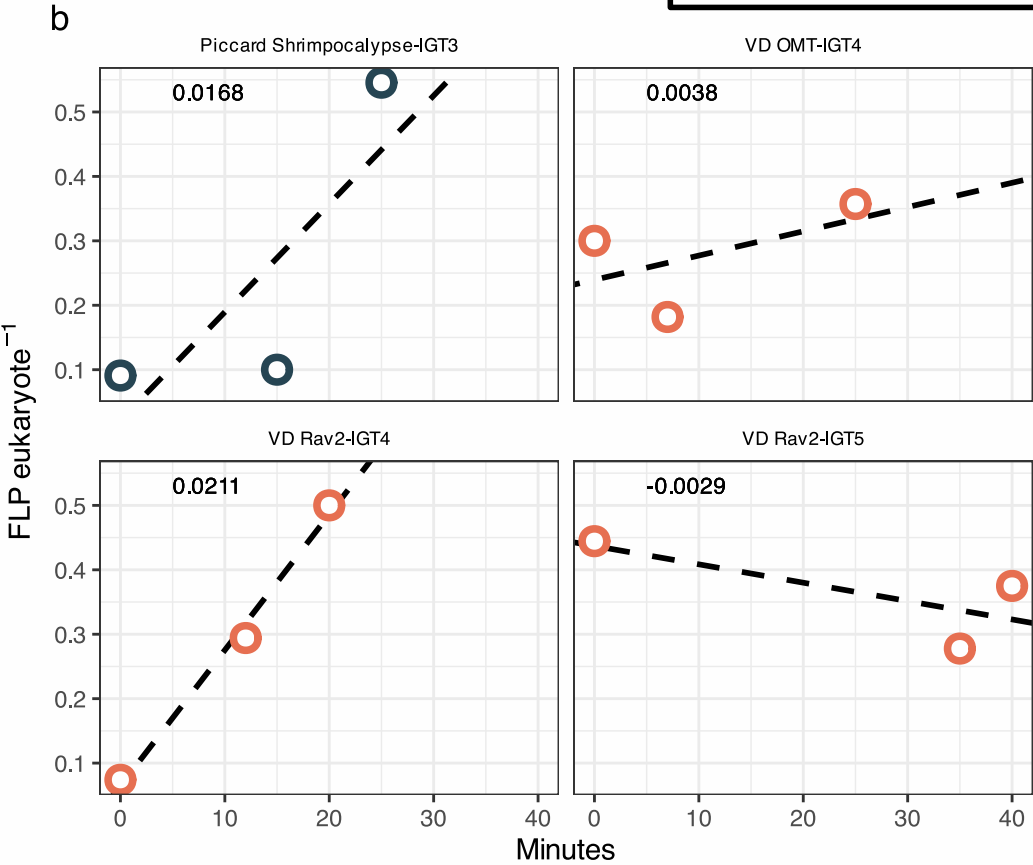

Supplement: SupplementaryInformation_wrae004 [file supplementaryinformation_wrae004.zip › figS4_new_wrae004.pdf]

Figure S5.

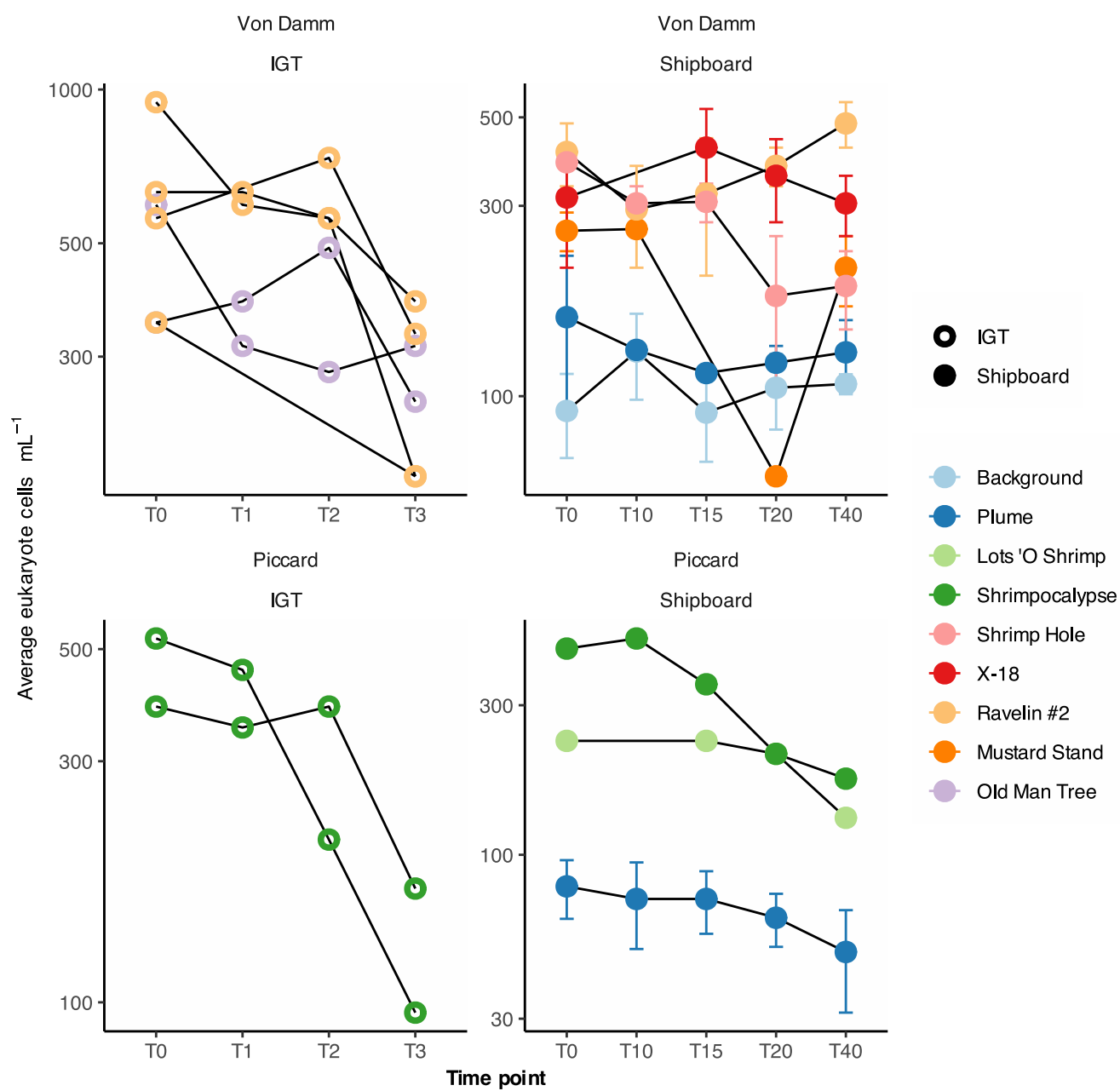

Supplement: SupplementaryInformation_wrae004 [file supplementaryinformation_wrae004.zip › figS5_new_wrae004.pdf]

Figure S6.

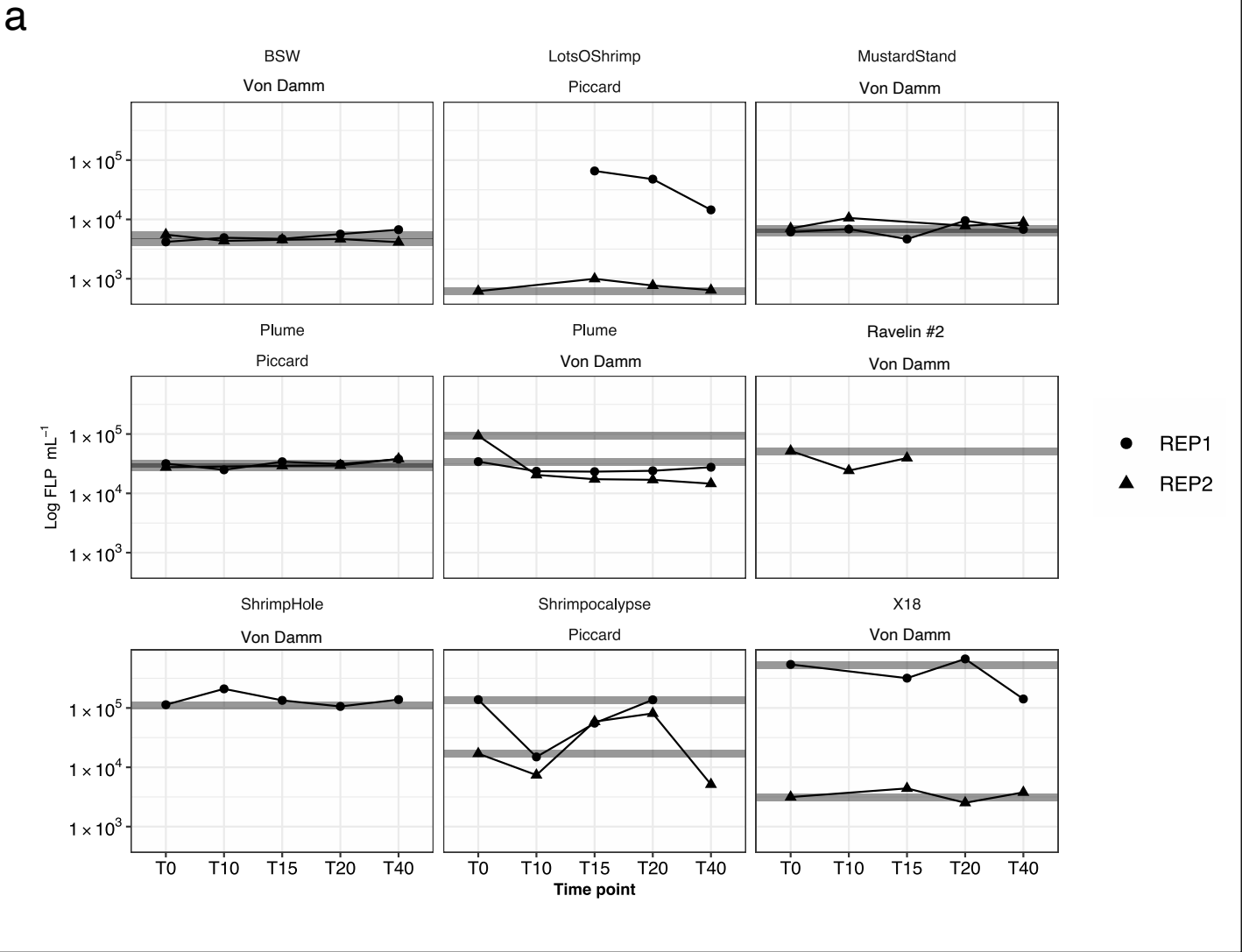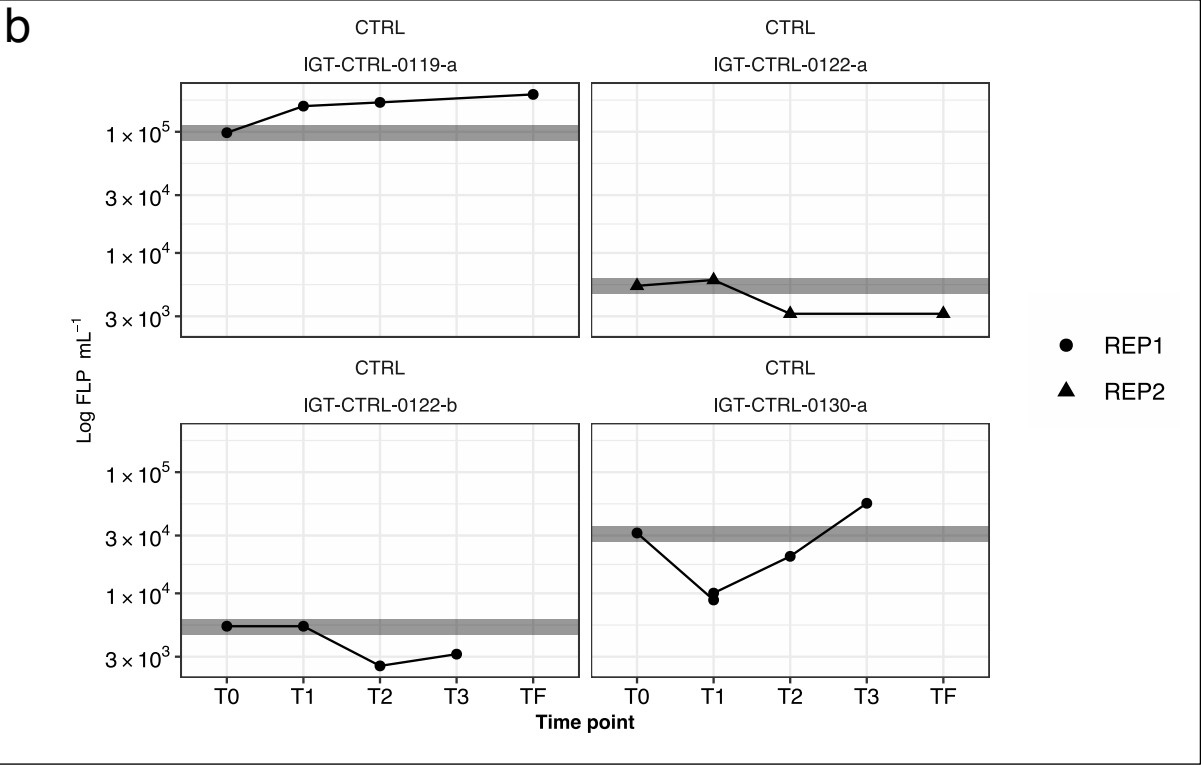

Supplement: SupplementaryInformation_wrae004 [file supplementaryinformation_wrae004.zip › figS6_new_wrae004.pdf]

Figure S7.

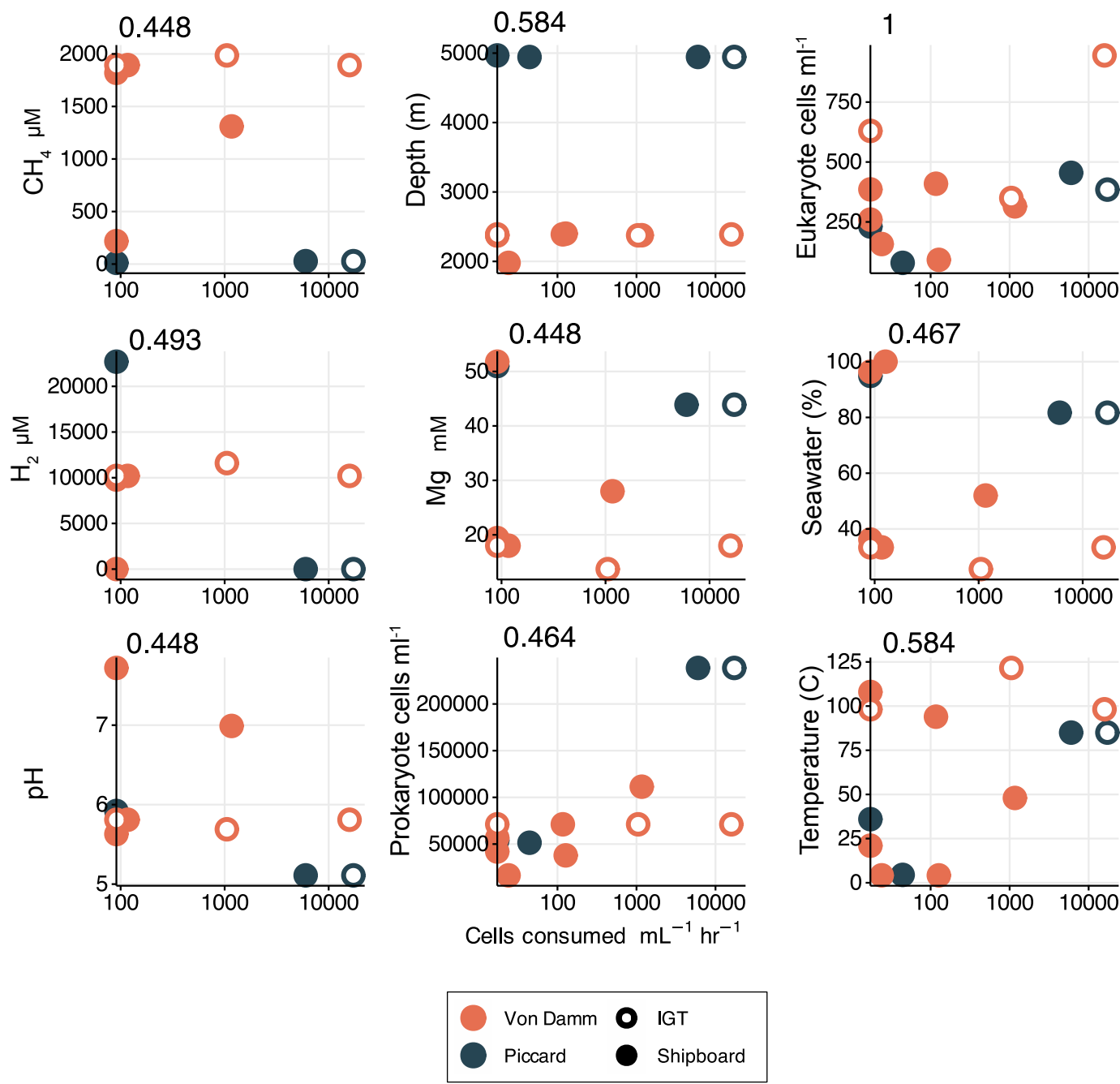

Supplement: SupplementaryInformation_wrae004 [file supplementaryinformation_wrae004.zip › figS7_new_lm_wrae004.pdf]

Figure S8.

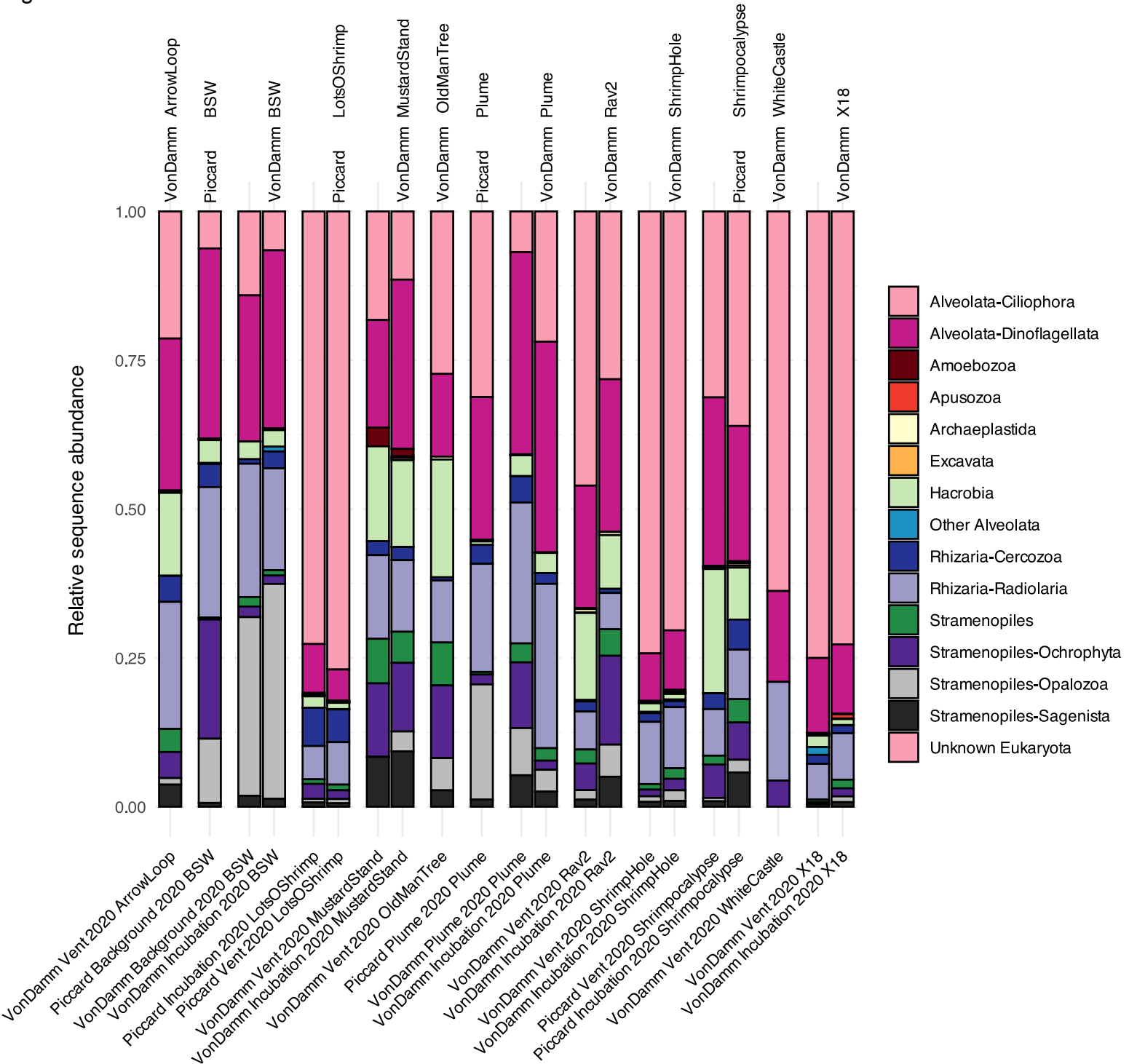

Supplement: SupplementaryInformation_wrae004 [file supplementaryinformation_wrae004.zip › figS8_new_wrae004.pdf]

Figure S9.

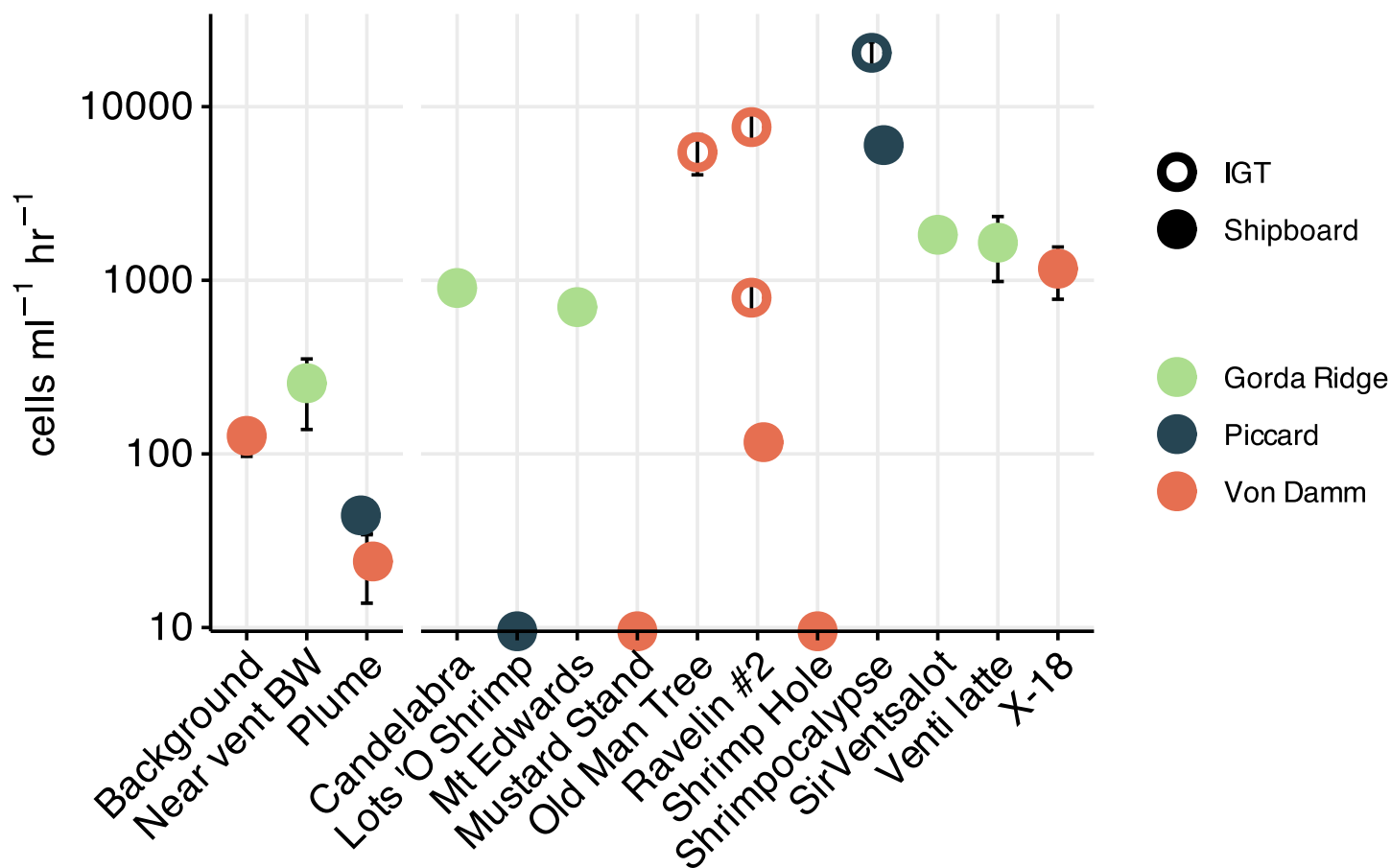

Supplement: SupplementaryInformation_wrae004 [file supplementaryinformation_wrae004.zip › figS9_new_wrae004.pdf]
